# Regional and global forcing of glacier retreat during the last deglaciation

**DOI:** 10.1038/ncomms9059

**Published:** 2015-08-21

**Authors:** Jeremy D. Shakun, Peter U. Clark, Feng He, Nathaniel A. Lifton, Zhengyu Liu, Bette L. Otto-Bliesner

**Affiliations:** 1 Department of Earth and Environmental Sciences, Boston College, Chestnut Hill, MA 02467, USA; 2 College of Earth, Ocean, and Atmospheric Sciences, Oregon State University, Corvallis, OR 97331, USA; 3 Center for Climatic Research, Nelson Institute for Environmental Studies, University of Wisconsin-Madison, Madison, WI 53706, USA; 4 Departments of Earth, Atmospheric, and Planetary Sciences and Physics and Astronomy, Purdue University, West Lafayette, IN 47907, USA; 5 Climate and Global Dynamics Division, National Center for Atmospheric Research, Boulder, CO 80307-3000, USA

## Abstract

The ongoing retreat of glaciers globally is one of the clearest manifestations of recent global warming associated with rising greenhouse gas concentrations. By comparison, the importance of greenhouse gases in driving glacier retreat during the most recent deglaciation, the last major interval of global warming, is unclear due to uncertainties in the timing of retreat around the world. Here we use recently improved cosmogenic-nuclide production-rate calibrations to recalculate the ages of 1,116 glacial boulders from 195 moraines that provide broad coverage of retreat in mid-to-low-latitude regions. This revised history, in conjunction with transient climate model simulations, suggests that while several regional-scale forcings, including insolation, ice sheets and ocean circulation, modulated glacier responses regionally, they are unable to account for global-scale retreat, which is most likely related to increasing greenhouse gas concentrations.

## Abstract (web summary)

The extent to which greenhouse gases forced glacier retreat during the last deglaciation remains unclear. Here, the authors recalculate cosmogenic nuclide ages for 195 glacier moraines and show that deglacial glacier retreat was broadly globally synchronous with rising levels of atmospheric CO_2_.

## Graphical abstract

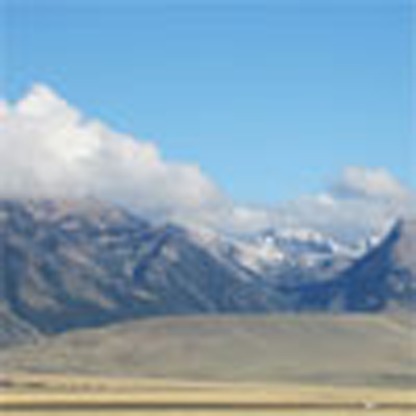

Glaciers are particularly sensitive to climate change, and their ongoing retreat globally is considered to be a robust signal of global warming over the last two centuries^1^. The previous episode of global glacier retreat occurred during the last deglaciation (∼19–11.5 ka), but dating uncertainties have prevented establishing whether it was regionally variable or globally synchronous during this period, leading to a range of explanations for retreat, including shifts in precipitation^2^, ocean circulation and the bipolar seesaw^3^,^4^, changes in the mean state of the tropical Pacific^5^, ice-sheet retreat^6^ and atmospheric water vapor content^7^. On the basis of a small sample of dated moraines, some studies suggested that CO_2_ forcing was important in causing retreat at certain times and across certain regions during the last deglaciation^8^,^9^, but it remains unclear as to what extent this and other forcings caused glacier retreat over the full deglaciation.

Here we use recent improvements in cosmogenic-nuclide production-rate calibrations to recalculate 1,116 cosmogenic (1,060 ^10^Be, 56 ^3^He) ages on 195 alpine moraines and 30 glaciated bedrock surfaces spanning Last Glacial Maximum (LGM) to modern ice extents to establish the timing of glacier fluctuations over the past 30,000 years (Supplementary Note 1). We find that there was regional variability in the timing of glacier fluctuations superimposed on a global pattern of broadly synchronous retreat that was largely coincident with the rise in CO_2_. Together with transient model simulations, our results suggest that greenhouse gases were the major driver of global-scale glacier retreat, while other factors modulated glacier responses regionally.

## Results

### Cosmogenic-nuclide data

**Figure 1:**
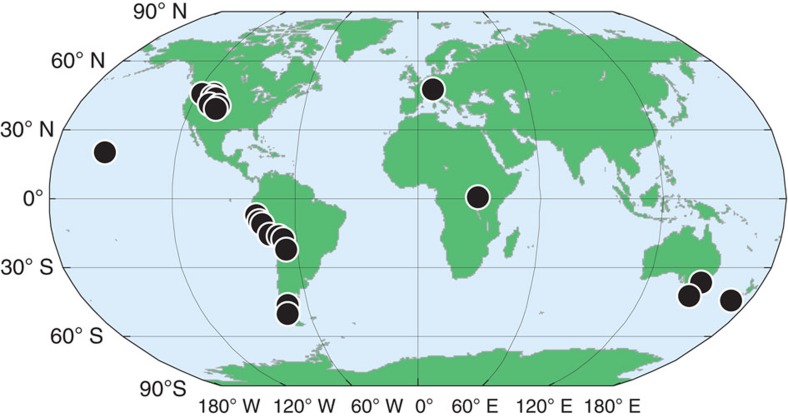
Study area locations. The locations of the 195 moraines and 30 glaciated bedrock surfaces.

Our moraine population extends from ∼55°S to ∼50°N, providing broad global coverage except at the high latitudes (Fig. 1). Earlier global ^10^Be production-rate calibrations^10^ appear to have been too high by 5–15% (refs 11, 12, 13, 14). Using a new, more accurate scaling model^15^ and recently published site production-rate estimates for ^10^Be and ^3^He, we developed a new global calibration for each nuclide, and recalculated all ages with updated MATLAB code derived from the CRONUS online calculator v 2.2 (ref. 10 and Lifton *et al*. ref. 15; see Methods section).

Given that the sizes and altitudes of our sampled glaciers varied considerably, we confine our analysis to valleys where the glacier terminus marking the local LGM has been dated, providing a common reference for comparing deglaciations from different regions. We then quantified glacier changes based on the horizontal and vertical components of their retreat relative to their LGM position. Reconstructed glaciers were scaled to units of normalized length and normalized elevation, from 1 at the position/elevation of their local LGM extent to 0 at their current terminus or cirque headwall position/elevation. These metrics likely do not scale in a simple, linear fashion with climate, but we suggest that they are sufficiently robust to capture the first-order patterns of glacier retreat, and consideration of both length and elevation changes helps to address hypsometric and geometric effects on glacier responses. For example, a comparison of these two metrics suggests a tendency for glacier termini from many regions to have initially experienced relatively large decreases in length as compared with small changes in elevation, followed by large changes in elevation associated with small-length changes (Supplementary Fig. 1). Because the sensitivity of glacier length to temperature is strongly influenced by slope^1^, we attribute this variation in response to the generally concave-up longitudinal profiles of the valleys occupied by the glaciers, resulting in lower glacier slopes at lower elevations as compared with higher elevations.

**Figure 2:**
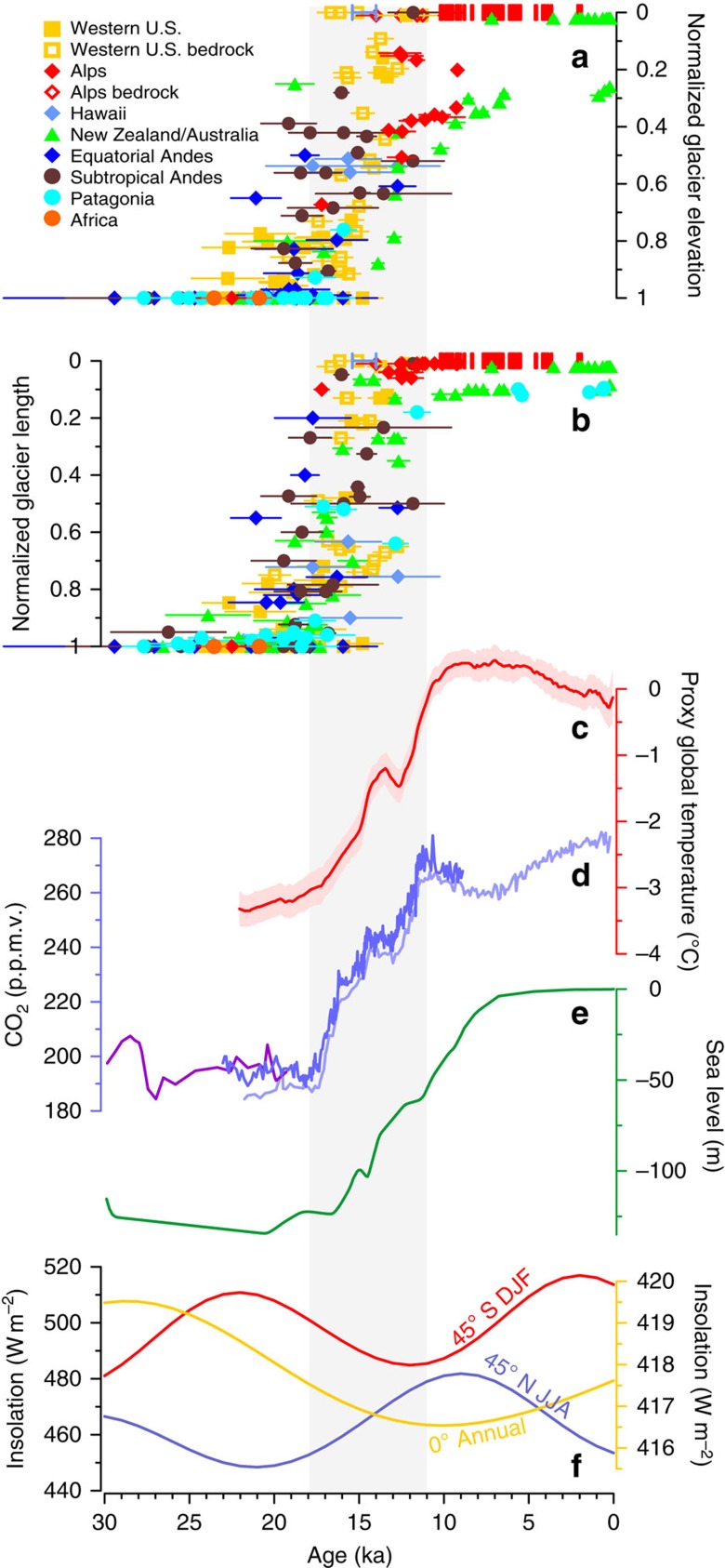
Glacier fluctuations and climate forcings. (**a** and **b**) Normalized moraine elevations and positions for the past 30 kyr. Closed symbols represent the mean of boulder surface-exposure ages on a moraine, and error bars (1σ) give the s.d. of the boulder ages plus the production-rate uncertainty, added in quadrature. Moraines are grouped by region. Open-colored symbols represent individual bedrock-exposure ages and 1σ external (analytical plus production rate) uncertainties. Red error bars in the Holocene at a length of 0 are radiocarbon and dendrochronologic ages from the Alps, and the blue error bar at 14.7 ka at length of 0 is a radiocarbon age for final deglaciation of Mauna Kea, Hawaii (Supplementary Data 1). (**c**) Proxy global temperature reconstruction^61^ (red). (**d**) Atmospheric CO_2_ from ice cores^62^,^63^,^64^ (blue and purple). (**e**) Global sea level^65^ (green). (**f**) Local summer insolation for 45°N (June-July-August, blue) and 45°S (December-January-February, red) and mean annual insolation for the equator (yellow)^66^. Gray vertical band highlights the interval of deglacial CO_2_ rise.

While there is some scatter in the resulting compilation of ages, Fig. 2 reveals that glaciers in our sample retreated in general synchrony between ∼19–11 ka. Some glaciers that appear to deglaciate anomalously early based on length, such as in the equatorial Andes and Alps, likely are overly influenced by their low slopes, and are in better agreement with the larger population when considering elevation (that is, relatively large reductions in length with correspondingly small changes in elevation). Likewise, Holocene glaciers in New Zealand and the subtropical Andes that span a large elevation range are quite small in length, and simply reflect recent retreat up steep valleys. In contrast, normalized glacier histories for regions that do not currently have ice (for example, Hawaii, much of the western United States) are effectively truncated at their cirque positions/elevations and cannot record climate fluctuations following local deglaciation. Moreover, retreat is more coherent in some regions (New Zealand, the western United States) than others (tropical South America), which may reflect differences in climatic variability, glacier sensitivities to climate change^16^, or geological scatter of the ages. In any case, the magnitude and spatial scale of this deglacial retreat is in marked contrast to the generally modest and more regional changes that occurred during the preceding and following ten millennia of the LGM and Holocene, respectively. Notably, this broadly synchronous retreat occurred despite glaciers spanning two orders of magnitude in length (10^0^–10^2^ km; Supplementary Fig. 2), 5 km in elevation, over 100° in latitude, various climatic settings^16^ and differing mass-balance regimes from mid-latitude glaciers with distinct mass-balance seasons to tropical glaciers that may accumulate and ablate all year.

Goodness-of-fit statistics between normalized glacier lengths and potential forcings are consistent with a primary role for CO_2_, suggesting that it can explain up to 81% of glacier variability over the past 30 kyr (Supplementary Fig. 3, Supplementary Note 2). In contrast, other possible drivers directly influencing glacier surface mass balance during the last deglaciation, such as insolation (Fig. 2f), ice sheets (Fig. 2e) and changes in ocean circulation, would have caused regionally and temporally variable responses (Supplementary Fig. 4), but cannot account for the near-global-scale timing established here. For example, we note that precessional forcing is opposite between the hemispheres, that increasing obliquity strengthened summers in the extratropics of both hemispheres but decreased mean annual insolation, and thus presumably ice ablation, in the tropics, and that these orbital parameters exhibited similarly large changes from 30–20 ka and 10–0 ka, yet glaciers were relatively stable during these times. Precipitation changes are similarly unlikely to account for global-scale retreat^1^, and in any case, the world probably became wetter on the whole rather than drier over the deglaciation^17^.

**Figure 3:**
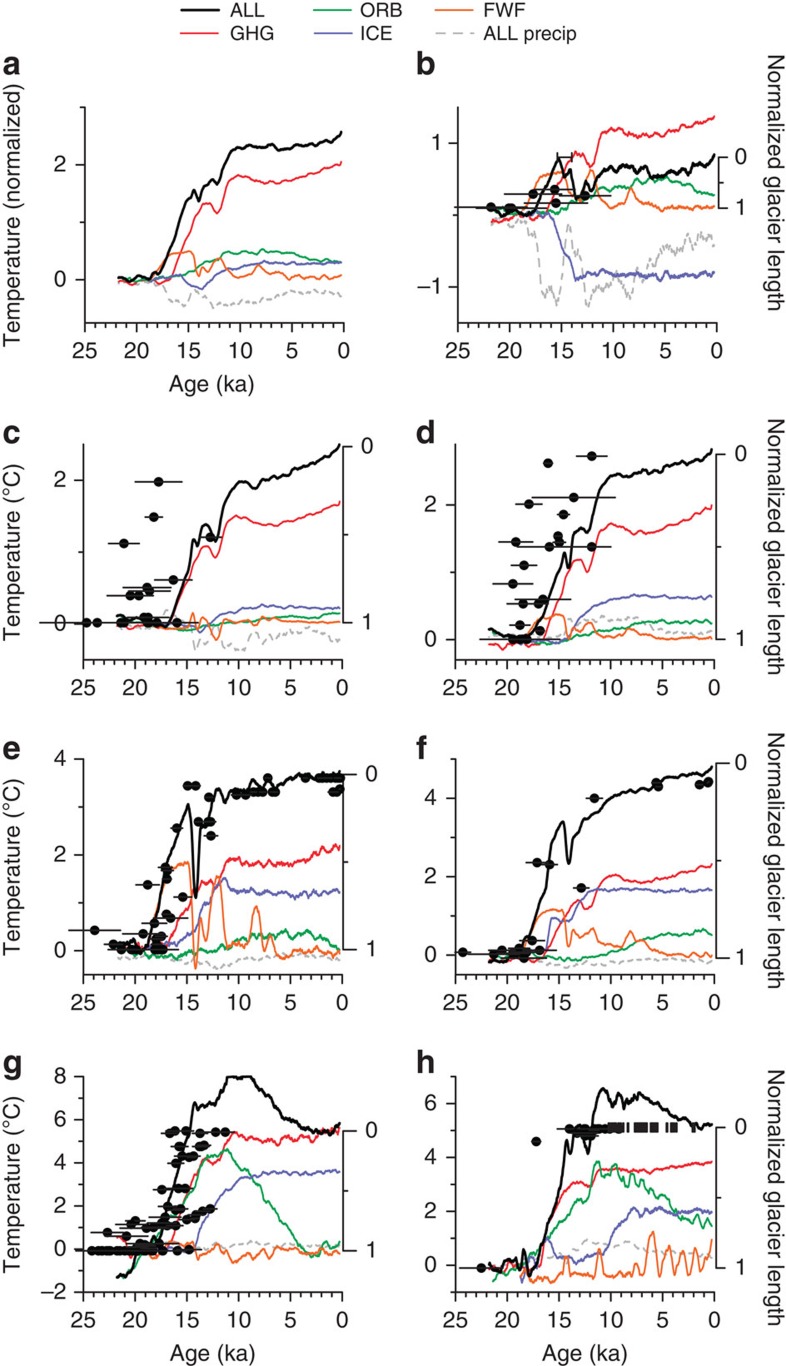
Modeled climate and reconstructed glacier fluctuations. Modeled temperatures from the single-forcing (colored lines) and ALL (black line) simulations, as well as normalized moraine positions (black dots) for (**b**) Hawaii, (**c**) tropical South America, (**d**) subtropical South America, (**e**) Australia and New Zealand, (**f**) Patagonia, (**g**) the western United States, and (**h**) the Alps. Error bars (1σ) give the s.d. of the boulder ages plus the production-rate uncertainty, added in quadrature. Local summer temperatures are shown for mid-latitude sites (June-July-August in Northern Hemisphere, December-January-February in Southern Hemisphere) and annual mean temperatures are shown for low-latitude sites. Modeled precipitation from the ALL simulation (gray dashed line) has been scaled to temperature as −25%=1 °C (ref. 1), and is shown for local winter at the mid-latitude sites and mean annual at the low-latitude sites. All model time series are 500-year moving averages and given as anomalies from 19 ka. The first panel (**a**) shows large-scale stacks for each simulation derived by averaging the regions together; the time series for each region were first normalized by the variance of their ALL simulation temperatures to give them equal weight in the stacks. *y*-axis on left of each graph is temperature and *y*-axis on right is normalized moraine position, which have been scaled to align maximum (1) and minimum (0) glacier extent with simulated Last Glacial Maximum and modern temperatures, respectively. See Supplementary Fig. 9 for analogous figure showing normalized moraine elevations.

While the normalized glacier length data exhibit scatter, several regional-scale features appear to be superimposed on the global signal of glacier retreat (Figs 2b and 3), possibly identifying an additional role for regional forcings. In particular, early retreat before the onset of the CO_2_ rise occurred in the western United States, the Andes and perhaps the Alps, Patagonia and Australia/New Zealand. Retreat in Hawaii was perhaps minimal until the Bølling^18^ (14.7 ka), and a second phase of retreat in New Zealand began at ∼13 ka (refs 19, 20).

### Transient climate modeling

We use transient simulations with a coupled global climate model (TraCE simulation^21^,^22^,^23^) to test the hypothesis that greenhouse gas forcing was the primary driver of global glacier retreat during the last deglaciation, modulated by regional variability associated with other forcing mechanisms. The simulations are driven by variations in individual forcing factors—greenhouse gases (GHG), insolation (ORB), ice sheets (ICE), and the Atlantic meridional overturning circulation (MOC)—as well as by all of these factors (ALL; Fig. 3). TraCE has been shown to replicate many key features of regional and global climate evolution during the last deglaciation^21^,^22^,^24^.

Temperature exerts a far stronger control on glacier surface mass balance than precipitation (we scale precipitation to temperature using the mass-balance approximation that a 25% precipitation increase compensates for a 1 °C warming^1^. This value has mostly been derived for mid-to-high-latitude glaciers, and it may be higher for low-latitude glaciers^16^, which would cause our scaling to overestimate the importance of precipitation changes in these areas) at all sites except Hawaii (Fig. 3), where precipitation variability may explain delayed glacier retreat or readvance during the Oldest Dryas (19–14.7 ka) and rapid deglaciation at the Bølling^18^. Simulated mean annual temperature time series at the locations of the tropical and subtropical moraines in the ALL and GHG runs are similar (Fig. 3c,d), indicating that CO_2_ was the major driver of ice loss in the low latitudes. Some moraines in these regions record large early responses (Fig. 3c,d), though they are primarily associated with glaciers that had low surface slopes and spanned a small elevation range (Supplementary Fig. 5), making them particularly sensitive to modest warming. Even still, there is a tendency for the central estimate of tropical terminal moraine ages to slightly precede the onset of the CO_2_ rise at 18 ka. Insofar as this region is strongly influenced by the El Niño-Southern Oscillation (ENSO), such an early timing of retreat might be associated with a change in ENSO variability^23^,^25^.

Much of the increase in simulated local summer temperatures at the Southern Hemisphere mid-latitude sites is associated with rising greenhouse gases, but warming prior to the CO_2_ rise can be attributed to the bipolar seesaw response in the MOC simulation^22^ (Fig. 3e,f), which would explain early glacier retreat at these locations^4^ (Supplementary Fig. 4). Subsequent variability in the MOC simulation explains cooling during the Antarctic Cold Reversal (14.6–12.8 ka), leading to glacier stabilization or readvance^19^,^20^. Additional warming at these sites in the ICE simulation may also reflect a bipolar seesaw response to weakening AMOC associated with ICE retreat (Supplementary Figs 4,6). Tropical and Southern Hemisphere responses are minor in the ORB simulation, suggesting insolation contributed little to deglaciation in these regions.

Greenhouse gas forcing remains important at Northern Hemisphere mid-latitude sites, but there is a comparable response in the ORB simulation, while warming associated with ice-sheet retreat is smaller in amplitude and later in phase (Fig. 3g,h). The warming from orbital forcing can explain the onset of glacier retreat in the western United States prior to CO_2_ rise (Fig. 3g). The dramatic retreat of ice in the Alps from its LGM position starting ∼17.2±1.3 ka to within kilometers of cirque headwalls by ∼15 ka, with a corresponding rise of the equilibrium line altitude of ∼500–600 m^26^, can be explained by the more than 5 °C of warming in the model simulation, about half of which is from greenhouse gas forcing (Fig. 3h). Licciardi *et al*.^6^ proposed that a large decrease in precipitation associated with the Oldest Dryas cold period may have induced glacier retreat, but when modeled local-winter precipitation is scaled to temperature, any such changes in the model are insignificant (Fig. 3h).

## Discussion

A reassessment of the cosmogenic-nuclide based chronology of glacier fluctuations spanning over 100° of latitude shows that glacier retreat was broadly synchronous with the increase in atmospheric CO_2_ and global temperature from 18–11 ka. Transient simulations with a coupled global climate model show that modulation by other forcings can explain regional variability in the glacier retreat chronology, with insolation explaining early deglaciation in the western United States, and seesaw responses to the AMOC explaining millennial variability in the Southern Hemisphere. Within dating uncertainties, onset of glacier retreat in the tropics is generally consistent with CO_2_ forcing, but the existing chronology cannot exclude earlier retreat, possibly identifying the influence of ENSO variability on glacier surface mass balance, or some other as yet unidentified regional forcing. While an imperfect comparison due to differences in time scales and several forcings, there is thus some similarity between glacier retreat over the last deglaciation and the last century. Both exhibit a globally coherent mode of retreat likely associated in large part with rising greenhouse gases, as well as regional variability in the pace and timing of retreat reflecting the operation of regional-scale forcings and heat redistributions within the climate system^27^,^28^.

**Figure 4:**
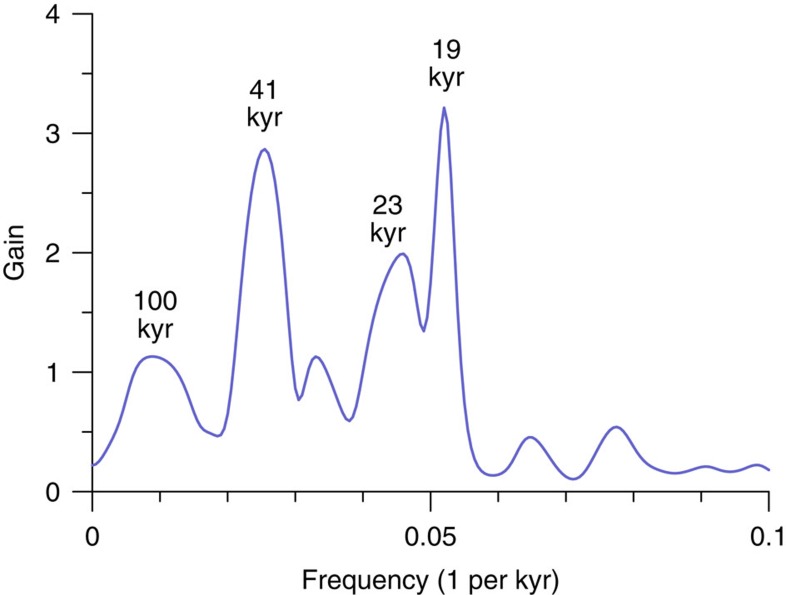
Ice volume-CO_2_ gain function. This plot shows the ratio of spectral power of an ice-volume reconstruction^67^ to the ice-core CO_2_ record^31^ over the past 800 kyr after normalizing each series to mean zero, unit variance. The periods of eccentricity (100 kyr), obliquity (41 kyr) and precession (19 and 23 kyr) are shown.

Our results have several potential implications. First, a longstanding puzzle concerns the quasi-uniform ∼1 km mountain snowline depression during the last glacial period despite presumably large differences in forcings and feedbacks around the world^29^. A primary control of glacier surface mass balance by CO_2_, however, would help explain this broadly homogenous pattern given the global forcing of greenhouse gases. Also, while polar amplification might be expected to yield larger snowline shifts at higher latitudes, the mid-to-low-latitude glaciers included in this study generally lie equatorward of the enhanced polar warming simulated by models^30^. Second, this large CO_2_-related snowline shift represents a considerable fraction of the thickness of the LGM ice sheets and, if it also occurred in the higher northern latitudes, would have likely had an important effect on ice-sheet surface mass balance. Modulation of ice-sheet response to orbital forcing by CO_2_ could explain why the ice sheets skipped orbital beats to exhibit 100-kyr power, since CO_2_ approached low values early in each glacial cycle^31^, as well as why terminations never occurred in the absence of strong greenhouse gas forcing during the past 800 kyr (ref. 31), and perhaps throughout the Quaternary^32^. A superposition of ice sheet responses to orbital and greenhouse gas forcing would also be consistent with the similar spectral power of ice volume and CO_2_ at the 100-kyr band, and enhanced ice-volume variability at the dominant precession and obliquity insolation periods during the last 800 kyr (Fig. 4). Third, our results highlight the sensitivity of alpine glaciers to a relatively modest CO_2_ increase (80 p.p.m.v.) and associated fast and slow feedbacks, supporting estimates of substantial mass loss from future deglaciation of global glaciers in response to projected increases in anthropogenic CO_2_ (ref. 33).

## Methods

### Recalibration of ^10^Be and ^3^He production rates

Accurate cosmogenic-nuclide production-rate estimates are critical for surface-exposure dating applications, particularly for global comparisons such as in this study. In addition to reliable calibrations of production rates at sites covering as wide a spatial and temporal range as possible, one needs consistent implementation of accurate production-rate scaling models to transform those rates to other sites of interest. The online calculator of Balco *et al*.^10^, is one of the most commonly used approaches for deriving ^10^Be-based exposure ages, using an internally consistent set of calculations for scaling models and site-specific production rates published at that time (2005 and earlier) to derive global-production-rate values and corresponding exposure ages. Goehring *et al*.^34^, used a modified version of the Balco *et al*.^10^, calculator to derive global-production rates for ^3^He. However, recent advances have pointed out shortcomings in previous scaling models, and more recent ^10^Be calibrations are yielding values that are consistently and significantly lower than those included in Balco *et al*.^10^. We briefly describe these advances below and incorporate them in a global recalibration for both ^10^Be and ^3^He production rates.

A new scaling model^15^ addresses significant biases in each of the scaling models included in Balco *et al*.^10^. These biases are particularly apparent at high-altitude and low-latitude locations—we refer the reader to Lifton *et al*.^15^, for details. This model, termed LSD, accurately reproduces the energy dependence of the atmospheric secondary cosmic-ray flux as a function of location and time, and enables the use of nuclide-specific scaling factors that account for differences in the energy-dependent production (and hence, scaling) of various *in situ* cosmogenic nuclides. All scaling calculations here were done using nuclide-specific formulations and the atmospheric, geomagnetic and solar framework considered in Lifton *et al*.^15^.

Lifton *et al*.^15^, and several previous scaling models used effective vertical cutoff rigidity (*R*_*C*_) to describe the dependence of the cosmic-ray flux on position within the geomagnetic field (including both dipolar and non-dipolar components). Cutoff rigidity is defined as the minimum rigidity (momentum per unit charge, *R*, usually measured in GV) that an incident primary cosmic-ray particle may possess and still be able to interact with the atmosphere at a given location (for example, see ref. 35); it is commonly limited to vertically incident particles for computational simplicity. *R*_*C*_ expands on that definition by accounting for the effects of the zone of alternating allowed and forbidden cosmic-ray trajectories near the Earth known as the penumbral region, which results from the interaction of complex, looping cosmic-ray trajectories with the solid Earth (for example, see ref. 36). However, O'Brien^37^ argues that neglecting non-vertically incident cosmic rays can underestimate appropriate cutoff values significantly, particularly at low latitudes (high *R*_*C*_). Calculating whole-sky cutoffs globally is prohibitively computationally expensive for our purposes (for example, see ref. 38), but Roesler *et al*.^39^, made use of a dipolar approximation that builds off of numerically calculated *R*_*C*_ values^36^. We follow Argento *et al*.^40^, and have incorporated this whole-sky cutoff rigidity approximation in our calculations for this study.

A number of high-quality ^10^Be and ^3^He production-rate calibrations have been published since Balco *et al*.^10^, and Goehring *et al*.^34^, We include 10 recent ^10^Be calibration studies^11^,^12^,^13^,^14^,^41^,^42^,^43^,^44^,^45^,^46^ in our ^10^Be analysis. We calculated sea-level and high-latitude reference production-rate values from each site with our new code, using the preferred sample selection of each study, and then calculated an arithmetic mean and s.d. from the results (Supplementary Table 1). As a whole, the new ^10^Be calibrations are much more consistent among the sites than the data set used in Balco *et al*.^10^, as well as, yielding mean values significantly lower (on the order of 10%) than those in the Version 2.2 update to the Balco *et al*.^10^, calculator (Supplementary Table 1). Of these, only the production-rate derived from Fenton *et al*.^41^, is significantly outside the group—Chauvenet's critierion indicates that it is an outlier—and we reject it from our preferred value for LSD scaling of 4.0±0.1 ^10^Be at g^−1^ per year (1σ). This value is similar to that arrived at by Heyman^47^ for time-dependent, Lal^48^/Stone^49^ scaling^10^, but we do not rely on arbitrarily rejecting individual sample outliers to reduce a particular site's statistical clustering (as measured by reduced *χ*^2^), or excluding sites with ambiguously defined ‘too large scatter' while including sites with much larger apparent scatter. We did, of course, focus solely on post-2005 calibration data sets, that is, those not contained within Balco *et al*.^10^, due to the overall consistency of the more recent data. We note that the Puerto Bandera moraines of Kaplan *et al*.^13^, were also included in the moraine data set of Ackert *et al*^50^. While certainly circular, excluding the Kaplan *et al*.^13^, data from the calibration has no effect on the resulting production rate—4.0±0.1 ^10^Be at g^−1^ per year with or without it.

We did a similar calculation for our ^3^He analysis, combining new data sets from Amidon and Farley^51^ (pyroxene only), Foeken *et al*.^52^, and Blard *et al*.^53^, with the data sets of Goehring *et al*.^34^ (Supplementary Table 2). The ^3^He data tend to be more scattered than the ^10^Be data set, both within and between sites, but all site results pass Chauvenet's criterion and thus are included. Grouping the data by study yields an arithmetic mean and s.d. for the sea-level and high-latitude ^3^He production rate for LSD scaling of 122±14 ^3^He at g^−1^ per year (1σ).

### Moraine ages

There is a considerable literature on how best to model moraine ages from individual boulder ages in the typical case that the scatter exceeds analytical uncertainty and thus must reflect geomorphic processes. Two competing processes are likely to dominate on deglacial-age moraines: prior exposure contributes inherited nuclides that lead to overestimates of moraine age, while boulder exhumation yields underestimates of the true moraine age. Applegate *et al*.^54^, suggested that moraines dominated by prior exposure will have boulder-age distributions skewed toward older ages, whereas incomplete exposure will skew the distribution toward younger ages. We find that the deviations of boulder ages from the mean age of each moraine used in this study are approximately evenly distributed about the mean (Supplementary Fig. 7). This lack of skewness suggests that errors due to prior and incomplete exposure may roughly cancel out in this global data set, even if they do not on individual moraines. We therefore report moraine ages as the arithmetic mean of boulder ages, and moraine age error bars as the s.d. of boulder ages plus the production-rate uncertainty, added in quadrature. We use 1σ production-rate uncertainties of 2.5% for ^10^Be and 11.5% for ^3^He. We excluded cosmogenic ages deemed outliers by the original authors (84 of 1,146). All exposure age calculations used the new ^10^Be and ^3^He calibrated global-production rates described above with nuclide-specific LSD scaling and the atmospheric, geomagnetic and solar framework described in Lifton *et al*.^15^. In addition to the cosmogenic ages, we also include radiocarbon dates on organic material recently uncovered by retreating glaciers in the Swiss^55^ and Austrian^56^ Alps, and a radiocarbon age for final deglaciation on Mauna Kea, Hawaii^57^. All data are provided in the Supplementary Data 1.

### Transient modeling

The four single-forcing transient simulations (GHG, ORB, ICE, MOC) of the TraCE simulations^22^ were conducted with the Community Climate System Model, version 3 to investigate the contribution of each individual climate forcing (greenhouse gases, insolation, ice sheets, Atlantic Meridional Overturning Circulation) to the modeled deglacial climate evolution in TraCE simulation ALL^21^. In this paper, the four single-forcing TraCE simulations are used to assess the individual contribution of the four climatic forcings to the regional and global signals of glacier retreat. As documented in the ‘Summary' section in Methods in ref. 22, all single-forcing transient simulations include dynamic vegetation feedback and a fixed annual cycle of aerosol forcing. Similar to simulation ALL, simulations ORB and GHG were branched off from an equilibrium LGM simulation^21^. Simulation ORB was forced only by transient variations of orbital configuration^58^ of the last 22 kyr, and simulation GHG was forced only by transient variations of greenhouse gas concentrations of the last 22 kyr^59^. All other forcing factors for simulations ORB and GHG are held constant with the values of 22 ka. Both simulations MOC and ICE were branched off at 19 ka from simulation ALL. Simulation MOC was forced only by transient variations of meltwater fluxes that were identical to those applied in simulation ALL^21^ (Supplementary Fig. 8). In simulation ICE, continental ice sheet orographies and extents were modified based on the time resolution of the ICE-5G (VM2) reconstruction^60^, that is, once per 1,000 years 19–16 ka, and once per 500 years from 16 ka onward. All other forcing factors for simulations MOC and ICE are held constant with the values of 19 ka.

## Additional information

**How to cite this article:** Shakun, J. D. *et al*. Regional and global forcing of glacier retreat during the last deglaciation. *Nat. Commun.* 6:8059 doi: 10.1038/ncomms9059 (2015).

## Supplementary Material

Supplementary Information Supplementary Figures 1-9, Supplementary Tables 1-2, Supplementary Notes 1-2 and Supplementary References

Supplementary Data 1 Cosmogenic surface exposure age data for all reconstructed glaciers

## References

[b1] Oerlemans J. Extracting a climate signal from 169 glacier records. Science 308, 675–677 (2005). 15746388 10.1126/science.1107046

[b2] Thackray G. D., Lundeen K. A. & Borgert J. A. Latest Pleistocene alpine glacier advances in the Sawtooth Mountains, Idaho, USA: reflections of midlatitude moisture transport at the close of the last glaciation. Geology 32, 225–228 (2004).

[b3] Young N. E., Briner J. P., Leonard E. M., Licciardi J. M. & Lee K. Assessing climatic and nonclimatic forcing of Pinedale glaciation and deglaciation in the western United States. Geology 39, 171–174 (2011).

[b4] Murray D. S. . Northern hemisphere forcing of the last deglaciation in southern Patagonia. Geology 40, 631–634 (2012).

[b5] Zech J., Zech R., May J.-H., Kubik P. W. & Veit H. Lateglacial and early Holocene glaciation in the tropical Andes caused by La Niña-like conditions. Palaeogeogr. Palaeoclimatol. Palaeoecol. 293, 248–254 (2010).

[b6] Licciardi J. M., Clark P. U., Brook E. J., Elmore D. & Sharma P. Variable responses of western US glaciers during the last deglaciation. Geology 32, 81–84 (2004).

[b7] Denton G. H. . Interhemispheric linkage of paleoclimate during the last glaciation. Geogr. Ann. A 81A, 107–153 (1999).

[b8] Schaefer J. M. . Near-synchronous interhemispheric termination of the last glacial maximum in mid-latitudes. Science 312, 1510–1513 (2006). 16763146 10.1126/science.1122872

[b9] Jomelli V. . A major advance of tropical Andean glaciers during the Antarctic cold reversal. Nature 513, 224–228 (2014). 25156258 10.1038/nature13546

[b10] Balco G., Stone J. O., Lifton N. A. & Dunai T. J. A complete and easily accessible means of calculating surface exposure ages or erosion rates from 10Be and 26Al measurements. Quat. Geochronol. 3, 174–195 (2008).

[b11] Balco G. . Regional beryllium-10 production rate calibration for late-glacial northeastern North America. Quat. Geochronol. 4, 93–107 (2009).

[b12] Putnam A. E. . *In situ* cosmogenic 10Be production-rate calibration from the Southern Alps, New Zealand. Quat. Geochronol. 5, 392–409 (2010).

[b13] Kaplan M. R. . *In-situ* cosmogenic 10Be production rate at Lago Argentino, Patagonia: implications for late-glacial climate chronology. Earth Planet Sci Lett 309, 21–32 (2011).

[b14] Goehring B. M. . Late glacial and holocene 10Be production rates for western Norway. J. Quat. Sci. 27, 89–96 (2012).

[b15] Lifton N., Sato T. & Dunai T. J. Scaling *in situ* cosmogenic nuclide production rates using analytical approximations to atmospheric cosmic-ray fluxes. Earth. Planet. Sci. Lett. 386, 149–160 (2014).

[b16] Sagredo E. A., Rupper S. & Lowell T. V. Sensitivities of the equilibrium line altitude to temperature and precipitation changes along the Andes. Quat. Res. 81, 355–366 (2014).

[b17] Clark P. U. . Global climate evolution during the last deglaciation. Proc. Natl Acad. Sci.USA 109, E1134–E1142 (2012). 22331892 10.1073/pnas.1116619109 PMC3358890

[b18] Anslow F. S., Clark P. U., Kurz M. D. & Hostetler S. W. Geochronology and paleoclimatic implications of the last deglaciation of the Mauna Kea Ice Cap, Hawaii. Earth. Planet. Sci. Lett. 297, 234–248 (2010).

[b19] Kaplan M. R. . Glacier retreat in New Zealand during the Younger Dryas stadial. Nature 467, 194–197 (2010). 20829791 10.1038/nature09313

[b20] Putnam A. E. . Glacier advance in southern middle-latitudes during the antarctic cold reversal. Nat. Geosci. 3, 700–704 (2010).

[b21] Liu Z. . Transient simulation of last deglaciation with a new mechanism for Bolling-Allerod warming. Science 325, 310–314 (2009). 19608916 10.1126/science.1171041

[b22] He F. . Northern hemisphere forcing of southern hemisphere climate during the last deglaciation. Nature 494, 81–85 (2013). 23389542 10.1038/nature11822

[b23] Liu Z. . Evolution and forcing mechanisms of El Nino over the past 21,000 years. Nature 515, 550–553 (2014). 25428502 10.1038/nature13963

[b24] Otto-Bliesner B. L. . Coherent changes of southeastern equatorial and northern African rainfall during the last deglaciation. Science 346, 1223–1227 (2014). 25477460 10.1126/science.1259531

[b25] Roe G. & O'Neal M. The response of glaciers to intrinsic climate variability: observations and models of late-Holocene variations in the Pacific Northwest. J. Glaciol. 55, 839–854 (2009).

[b26] Ivy-Ochs S., Kerschner H., Kubik P. W. & Schlüchter C. Glacier response in the European Alps to Heinrich Event 1 cooling: the Gschnitz stadial. J. Quat. Sci. 21, 115–130 (2006).

[b27] Bindoff N. L. . Detection and Attribution of Climate Change: from Global to Regional 867–952 (Cambridge University Press (2013).

[b28] Marzeion B., Cogley J. G., Richter K. & Parkes D. Attribution of global glacier mass loss to anthropogenic and natural causes. Science 345, 919–921 (2014). 25123485 10.1126/science.1254702

[b29] Broecker W. S. & Denton G. H. The role of ocean-atmosphere reorganizations in glacial cycles. Geochim. Cosmochim. Acta. 53, 2465–2501 (1989).

[b30] Pithan F. & Mauritsen T. Arctic amplification dominated by temperature feedbacks in contemporary climate models. Nat. Geosci. 7, 181–184 (2014).

[b31] Luthi D. . High-resolution carbon dioxide concentration record 650,000-800,000[thinsp]years before present. Nature 453, 379–382 (2008). 18480821 10.1038/nature06949

[b32] Herbert T. D., Peterson L. C., Lawrence K. T. & Liu Z. Tropical Ocean Temperatures Over the Past 3.5 Million Years. Science 328, 1530–1534 (2010). 20558711 10.1126/science.1185435

[b33] Marzeion B., Jarosch A. H. & Hofer M. Past and future sea-level change from the surface mass balance of glaciers. Cryosphere 6, 1295–1322 (2012).

[b34] Goehring B. M. . A reevaluation of in situ cosmogenic 3He production rates. Quat. Geochronol. 5, 410–418 (2010).

[b35] Cooke D. J. . On cosmic-ray cut-off terminology. Il Nuovo Cimento C 14, 213–234 (1991).

[b36] Shea M. A., Smart D. F. & McCracken K. G. A study of vertical cutoff rigidities using sixth degree simulations of the geomagnetic field. J. Geophys. Res. 70, 4117–4130 (1965).

[b37] O'Brien K. Limitations of the use of the vertical cut-off to calculate cosmic-ray propagation in the Earth's atmosphere. Radiat. Prot. Dosimetry 128, 259–260 (2008). 18346965 10.1093/rpd/ncn062

[b38] Clem J. M. . Contribution of obliquely incident particles to neutron monitor counting rate. J. Geophys. Res.: Space Phys. 102, 26919–26926 (1997).

[b39] Roesler S., Heinrich W. & Schraube H. Calculation of radiation fields in the atmosphere and comparison to experimental data. Radiat. Res. 149, 87–97 (1998). 9421158

[b40] Argento D. C., Stone J. O., Reedy R. C. & O'Brien K. Physics-based modeling of cosmogenic nuclides part I–radiation transport methods and new insights. Quat. Geochronol. 26, 29–43 (2014).

[b41] Fenton C. R. . Regional 10Be production rate calibration for the past 12 ka deduced from the radiocarbon-dated Grøtlandsura and Russenes rock avalanches at 69° N, Norway. Quat. Geochronol 6, 437–452 (2011).

[b42] Claude A. . The Chironico landslide (Valle Leventina, southern Swiss Alps): age and evolution. Swiss J. Geosci. 107, 273–291 (2014).

[b43] Ballantyne C. K. & Stone J. O. Did large ice caps persist on low ground in north-west Scotland during the Lateglacial Interstade? J. Quat. Sci. 27, 297–306 (2012).

[b44] Lifton N. . *In situ* cosmogenic nuclide production rate calibration for the CRONUS-Earth project from Lake Bonneville, Utah, shoreline features. Quat. Geochronol. 26, 56–59 (2015).

[b45] Kelly M. A. . A locally calibrated, late glacial 10Be production rate from a low-latitude, high-altitude site in the Peruvian Andes. Quat. Geochronol. 26, 70–85 (2013).

[b46] Young N. E., Schaefer J. M., Briner J. P. & Goehring B. M. A 10Be production-rate calibration for the Arctic. J. Quat. Sci. 28, 515–526 (2013).

[b47] Heyman J. Paleoglaciation of the Tibetan Plateau and surrounding mountains based on exposure ages and ELA depression estimates. Quat. Sci. Rev. 91, 30–41 (2014).

[b48] Lal D. Cosmic ray labeling of erosion surfaces: *in-situ* nuclide production rates and erosion models. Earth. Planet. Sci. Lett. 104, 424–439 (1991).

[b49] Stone J. Air pressure and cosmogenic isotope production. J. Geophys. Res. 105, 23753–23759 (2000).

[b50] Ackert R. P. Jr. . Patagonian Glacier response during the late Glacial-Holocene transition. Science 321, 392–395 (2008). 18635799 10.1126/science.1157215

[b51] Amidon W. H. & Farley K. A. Cosmogenic 3He production rates in apatite, zircon and pyroxene inferred from Bonneville flood erosional surfaces. Quat. Geochronol. 6, 10–21 (2011).

[b52] Foeken J. P. T., Stuart F. M. & Mark D. F. Long-term low latitude cosmogenic 3He production rate determined from a 126 ka basalt from Fogo, Cape Verdes. Earth. Planet. Sci. Lett. 359–360, 14–25 (2012).

[b53] Blard P. H. . Cosmogenic 3He production rate in the high tropical Andes (3800, m, 20°S): Implications for the local last glacial maximum. Earth. Planet. Sci. Lett. 377–378, 260–275 (2013).

[b54] Applegate P. J., Urban N. M., Laabs B. J. C., Keller K. & Alley R. B. Modeling the statistical distributions of cosmogenic exposure dates from moraines. Geosci. Model Dev. 3, 293–307 (2010).

[b55] Joerin U. E., Stocker T. F. & Schlüchter C. Multi century glacier fluctuations in the Swiss Alps during the Holocene. Holocene 16, 697–704 (2006).

[b56] Nicolussi K. & Patzelt G. Discovery of early Holocene wood and peat on the forefield of the Pasterze Glacier, Eastern Alps, Austria. Holocene 10, 191–199 (2000).

[b57] Peng L. & King J. W. A late quaternary geomagnetic secular variation record from Lake Waiau, Hawaii, and the question of the Pacific nondipole low. J. Geophys. Res. 97, 4407–4424 (1992).

[b58] Berger A. L. Long-term variations of daily insolation and quaternary climatic changes. J. Atmos. Sci. 35, 2362–2367 (1978).

[b59] Joos F. & Spahni R. Rates of change in natural and anthropogenic radiative forcing over the past 20,000 years. Proc. Natl Acad. Sci. USA 105, 1425–1430 (2008). 18252830 10.1073/pnas.0707386105 PMC2234160

[b60] Peltier W. R. Global glacial isostasy and the surface of the ice-age earth: the ice-5G (VM2) model and grace. Annu. Rev. Earth. Planet Sci. 32, 111–149 (2004).

[b61] Shakun J. D. . Global warming preceded by increasing carbon dioxide concentrations during the last deglaciation. Nature 484, 49–54 (2012). 22481357 10.1038/nature10915

[b62] Ahn J. & Brook E. J. Atmospheric CO2 and climate from 65 to 30 ka B.P. Geophys. Res. Lett. 34, (2007).

[b63] Monnin E. . Atmospheric CO_2_ concentrations over the last glacial termination. Science 291, 112–114 (2001). 11141559 10.1126/science.291.5501.112

[b64] Marcott S. A. . Centennial-scale changes in the global carbon cycle during the last deglaciation. Nature 514, 616–619 (2014). 25355363 10.1038/nature13799

[b65] Lambeck K., Rouby H., Purcell A., Sun Y. & Sambridge M. Sea level and global ice volumes from the last glacial maximum to the Holocene. Proc. Natl Acad. Sci. USA 111, 15296–15303 (2014). 25313072 10.1073/pnas.1411762111 PMC4217469

[b66] Laskar J. . A long term numerical solution for the insolation quantities of the Earth. Astron. Astrophys. 428, 261–285 (2004).

[b67] Bintanja R., van de Wal R. S. W. & Oerlemans J. Modelled atmospheric temperatures and global sea levels over the past million years. Nature 437, 125–128 (2005). 16136140 10.1038/nature03975

