# Supplementary material for: Regional and global forcing of glacier retreat during the last deglaciation
Source: Nat Commun. 2015 Aug 21;6:8059. doi: 10.1038/ncomms9059 (PMC4560787; doi:10.1038/ncomms9059)
Supplement: Supplementary Information — Supplementary Figures 1-9, Supplementary Tables 1-2, Supplementary Notes 1-2 and Supplementary References [file ncomms9059-s1.pdf]

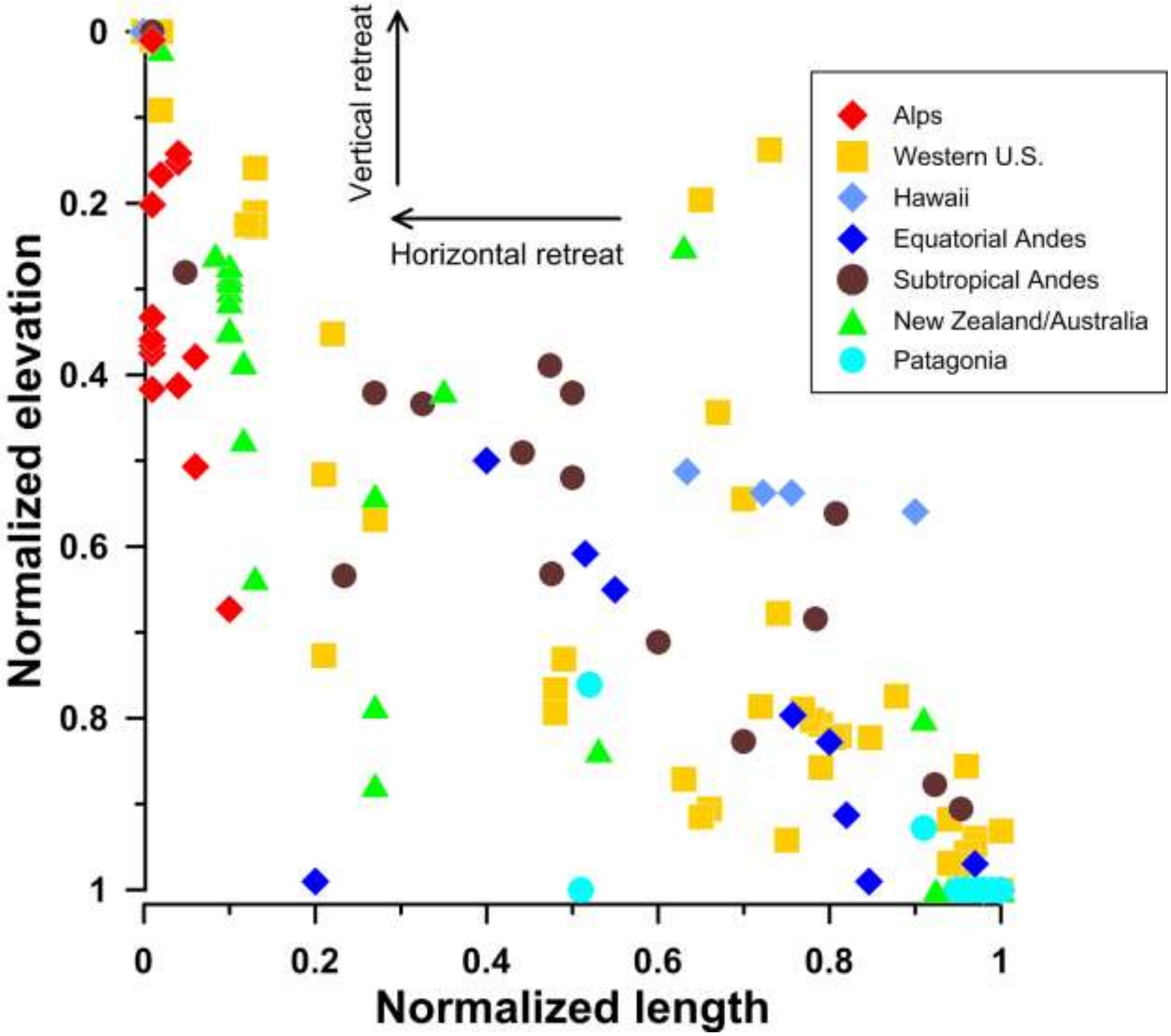

Supplementary Figure 1. Normalized glacier terminus elevation changes versus normalized glacier length changes.

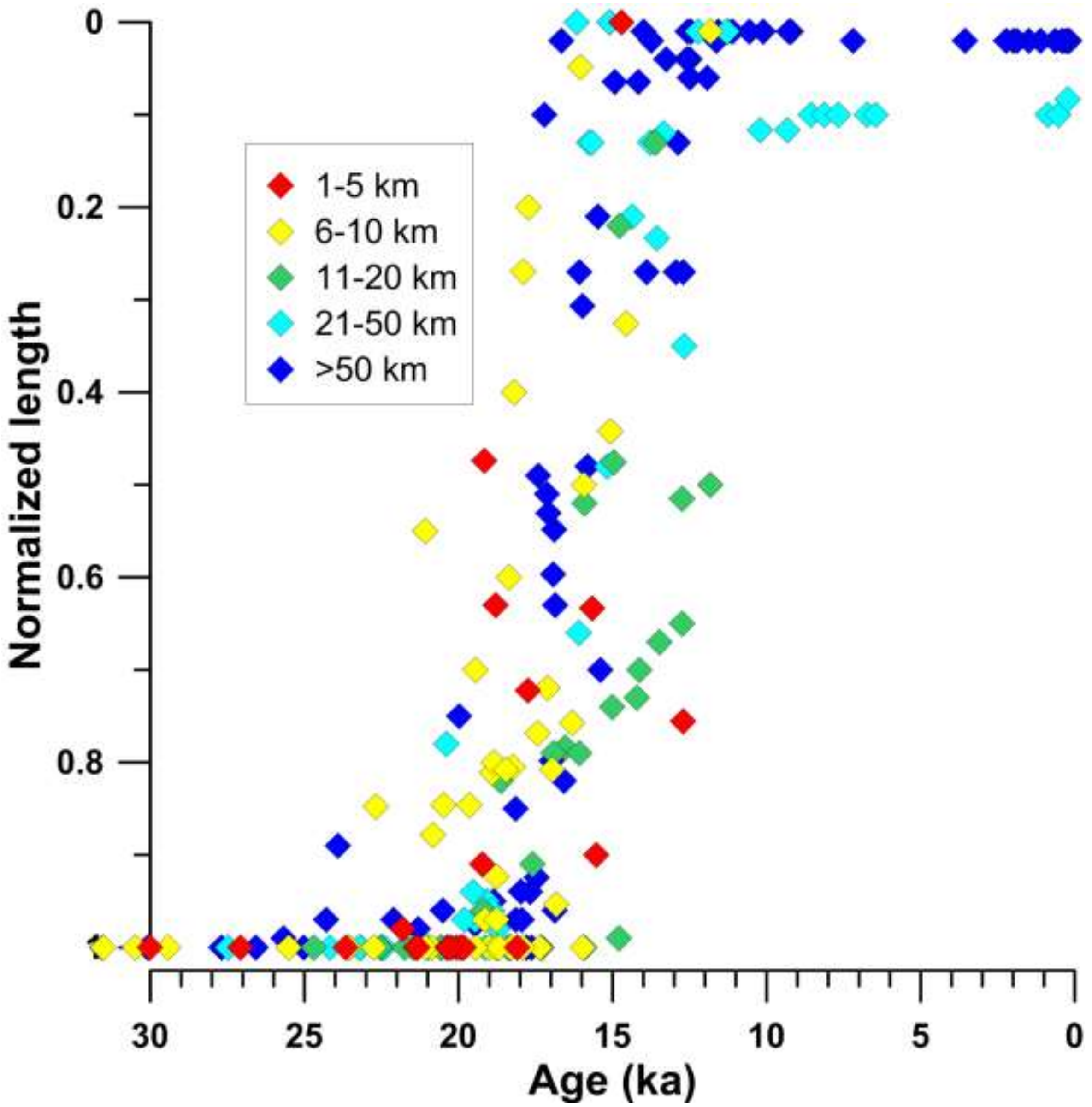

Supplementary Figure 2. Normalized glacier lengths through time, as shown in Fig. 2 in the main text, but divided into several size classes based upon their LGM extent.

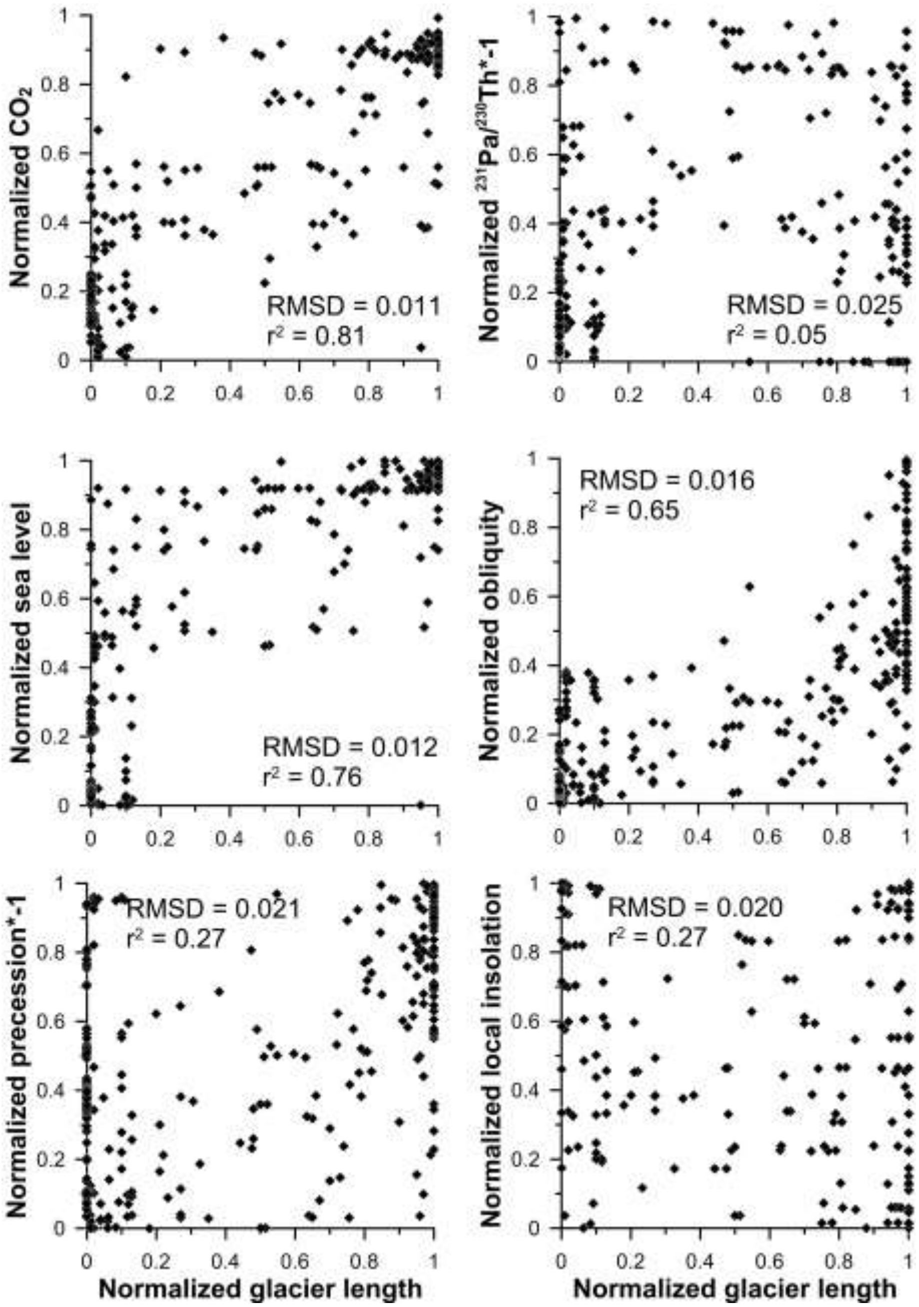

Supplementary Figure 3. Scatterplots of normalized glacier lengths (x-axes) versus normalized forcings (y-axes) showing root-mean-squared deviation (RMSD) and coefficient of determination (r^2). Forcings considered include CO_2 (ref. [1]), AMOC variations as represented by a Pa/Th record[2], ice sheets as represented by eustatic sea level[3], obliquity, precession, and local insolation (JJA for northern extratropical sites, DJF for southern extratropical sites, mean annual for tropical sites).

ALL simulation

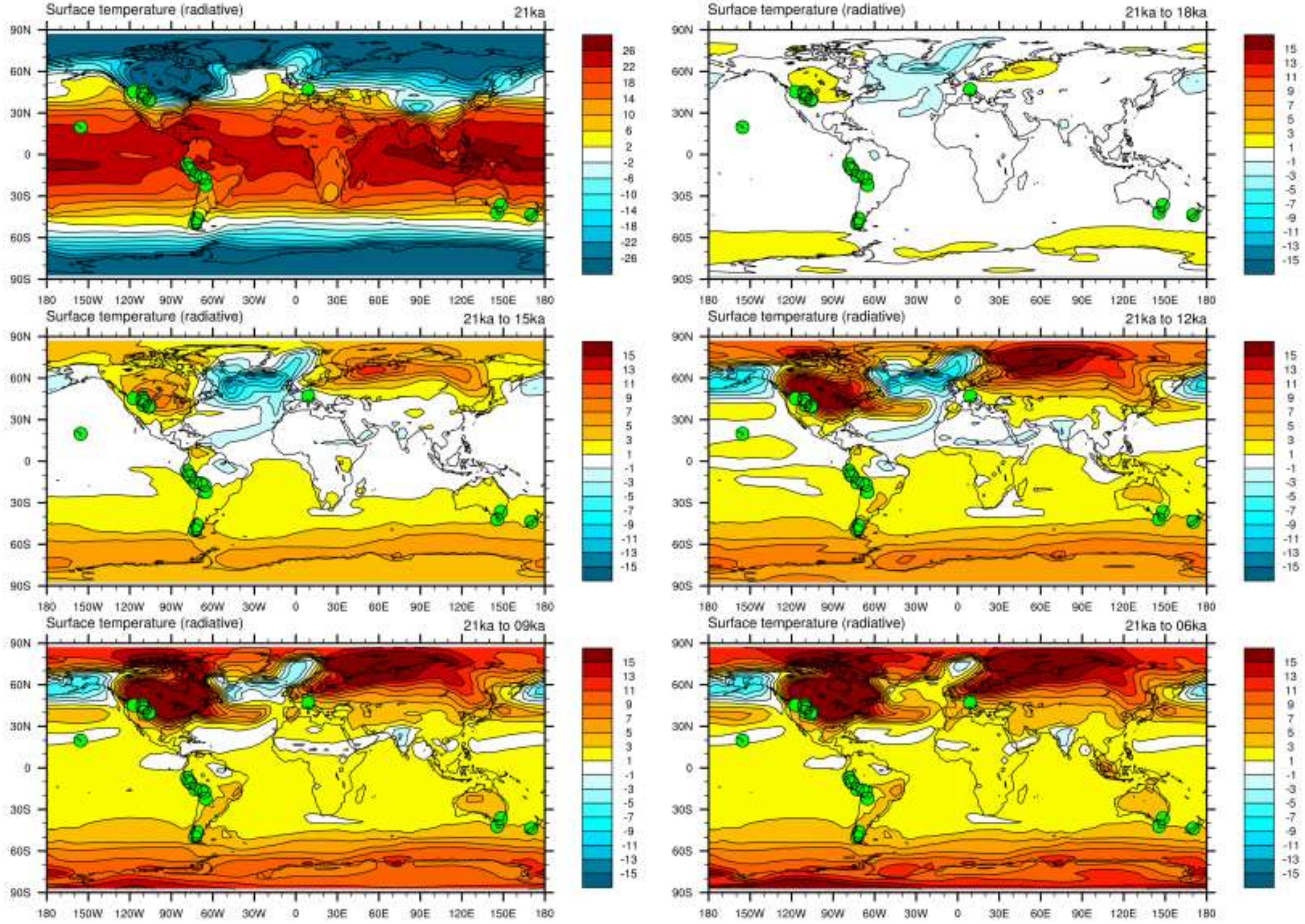

GHG simulation

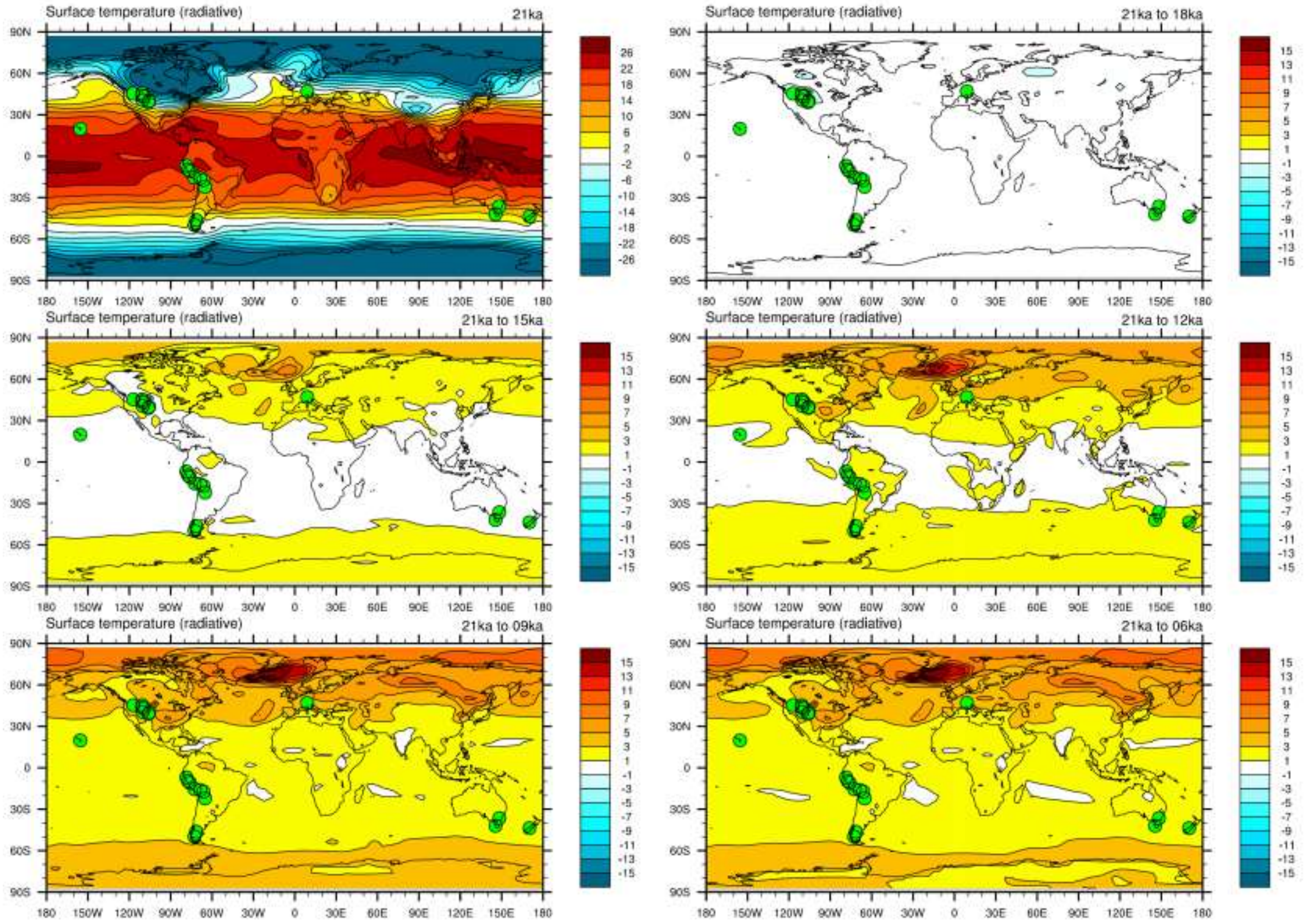

ORB simulation

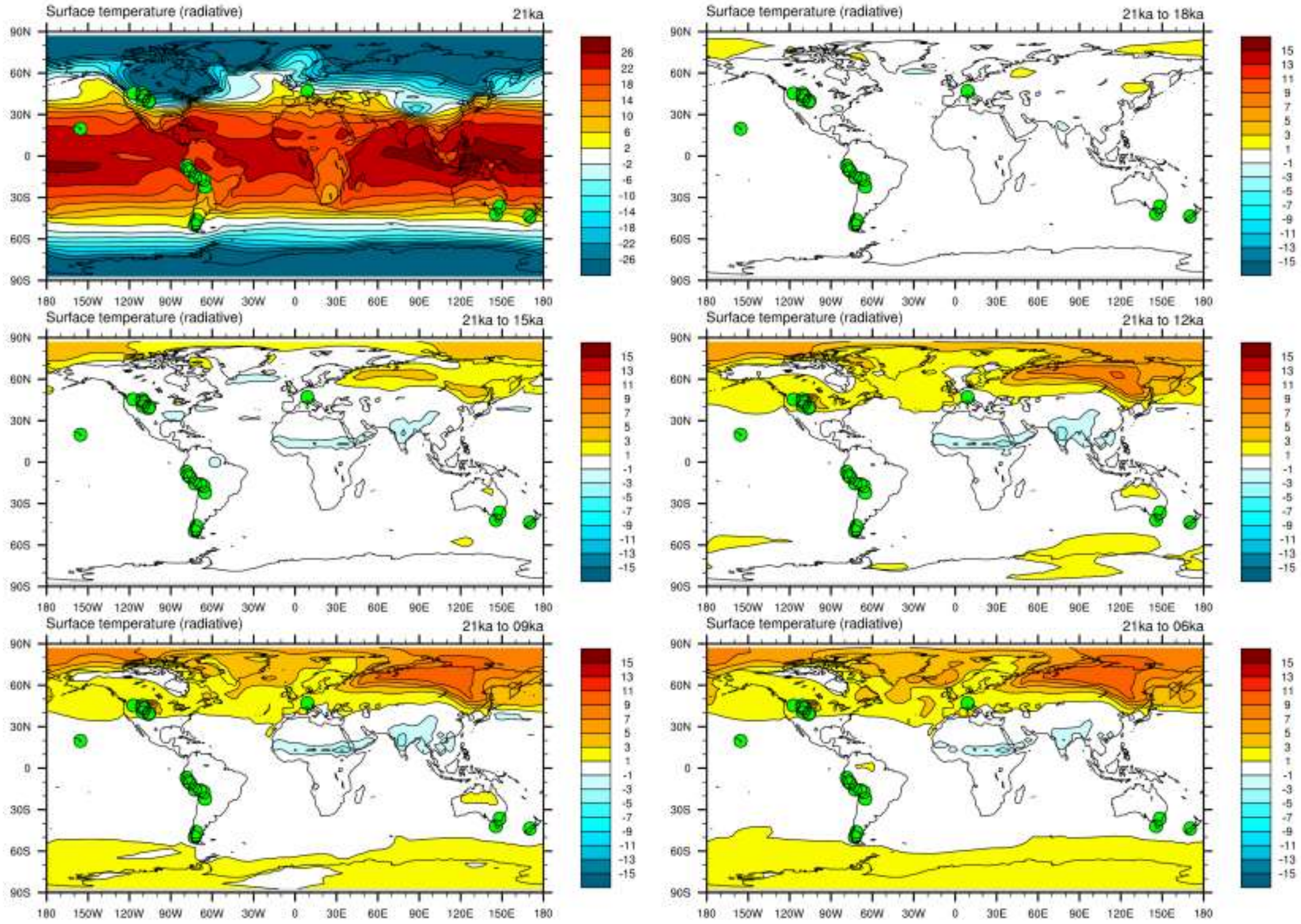

ICE simulation

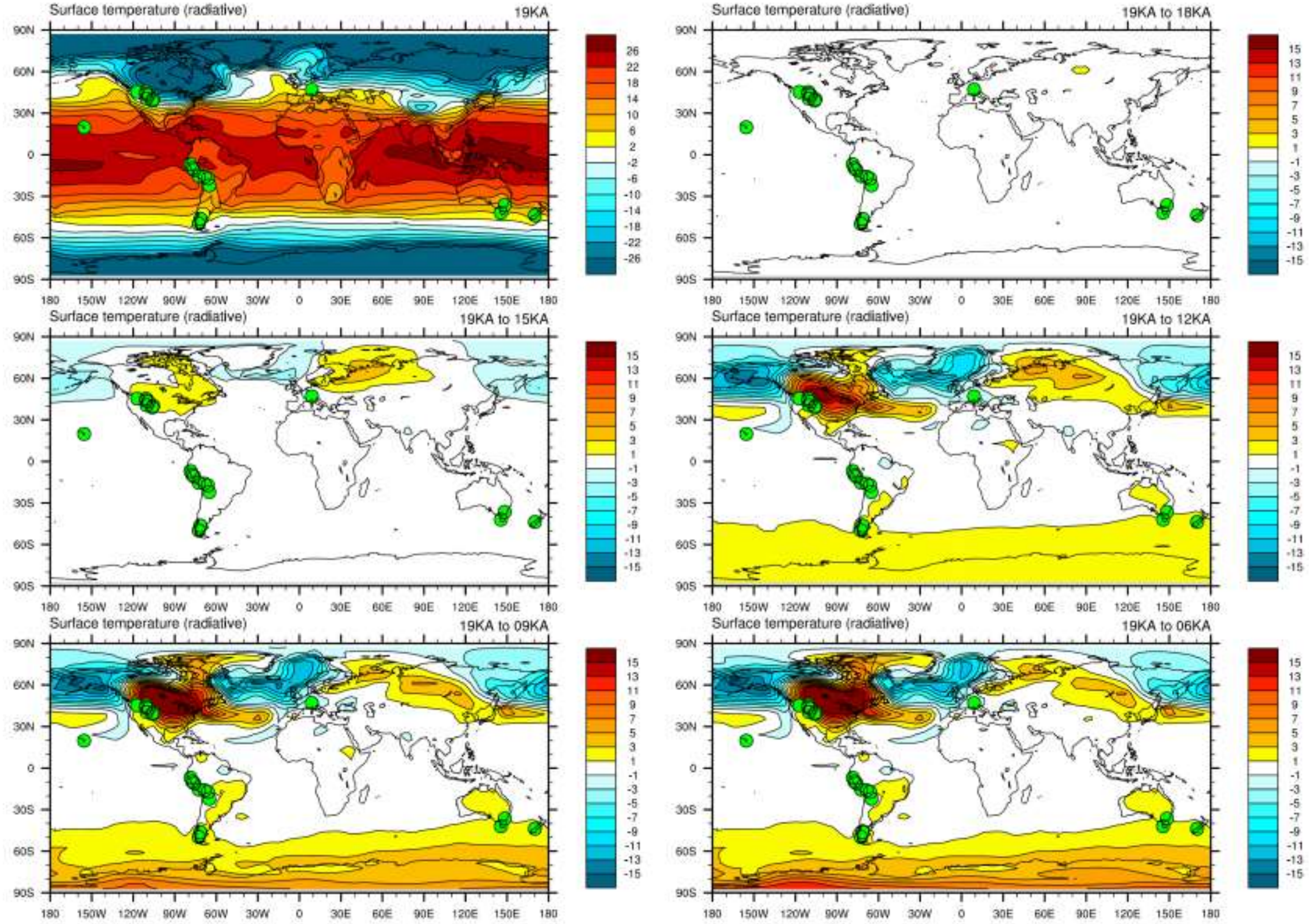

MOC simulation

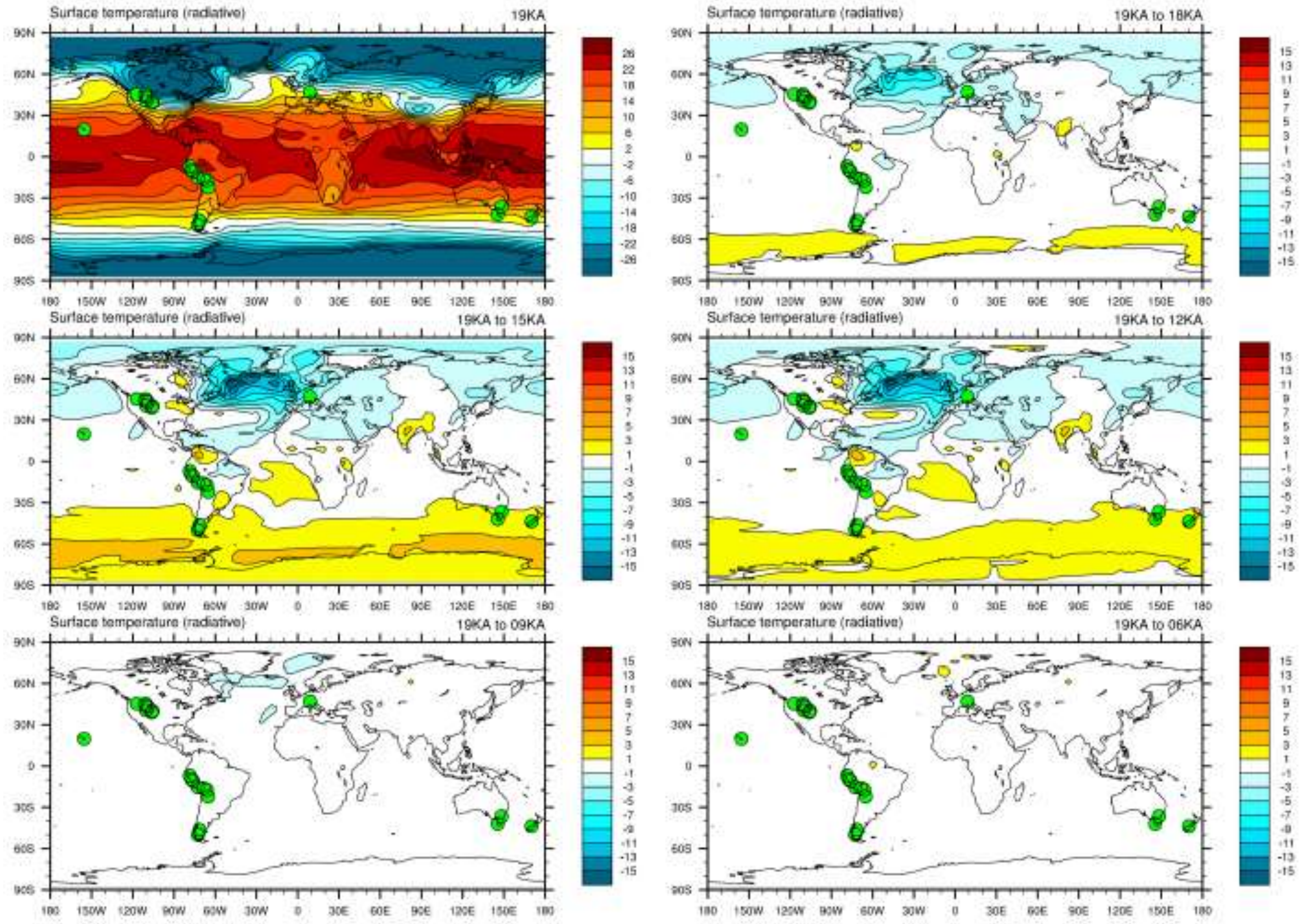

Supplementary Figure 4. Each figure above shows mean annual surface temperature at the Last Glacial Maximum (LGM; 21 ka for simulation ALL, GHG, and ORB, and 19 ka for simulation ICE and MOC) in the upper left panel for the various transient simulations, followed by surface temperature anomalies from the LGM at 18, 15, 12, 9, and 6 ka. Light blue dots on each map represent the locations of the glacial moraines synthesized in this study. Units are °C.

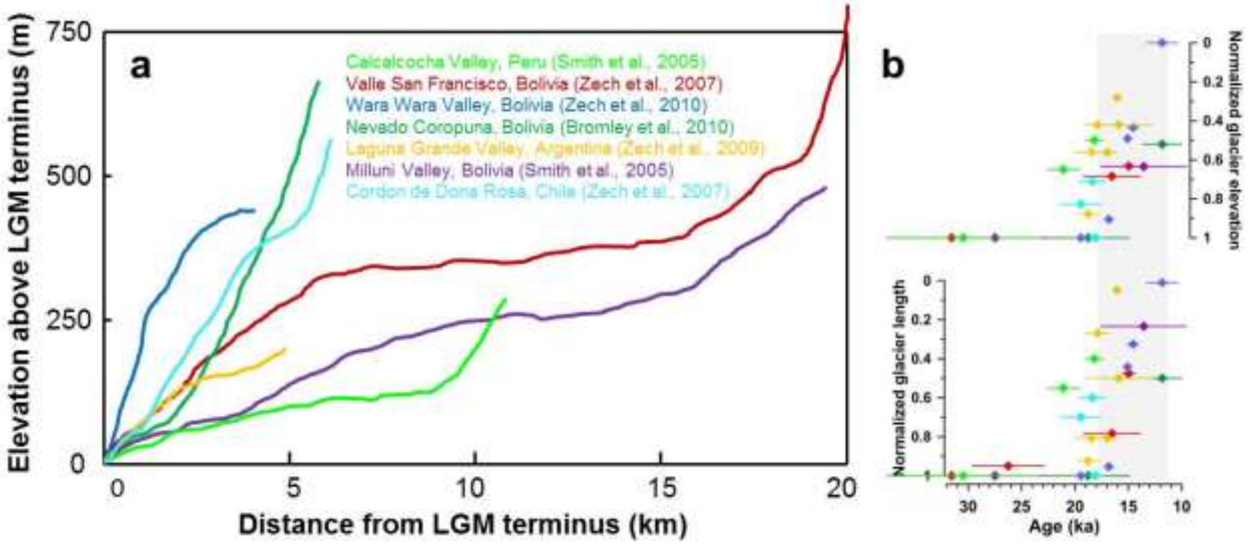

Supplementary Figure 5. (**a**) Valley profiles for several subtropical and equatorial Andes sites between the local LGM moraine and cirque headwall or modern glacier terminus. (**b**) The corresponding normalized glacier length and elevation histories for these sites. Gray bar highlights deglacial CO_2 and global temperature rise.

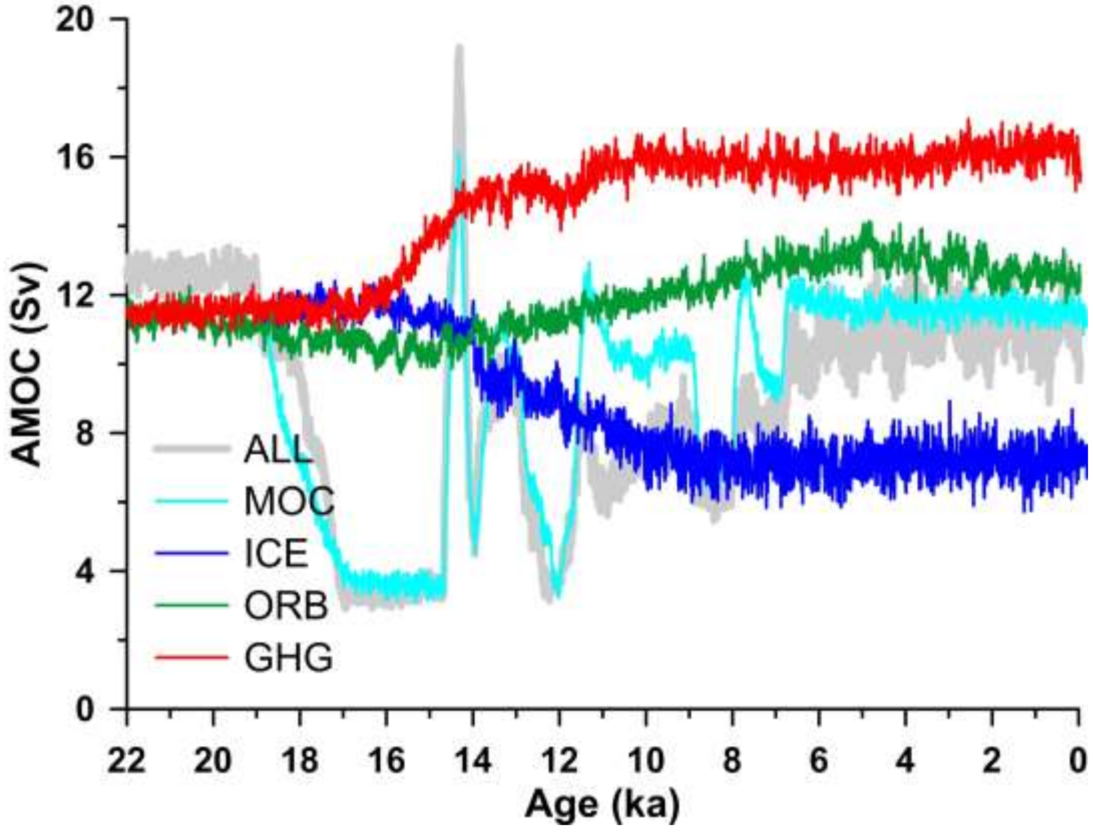

Supplementary Figure 6. Atlantic Meridional Overturning Circulation (AMOC) strength in the transient model simulations.

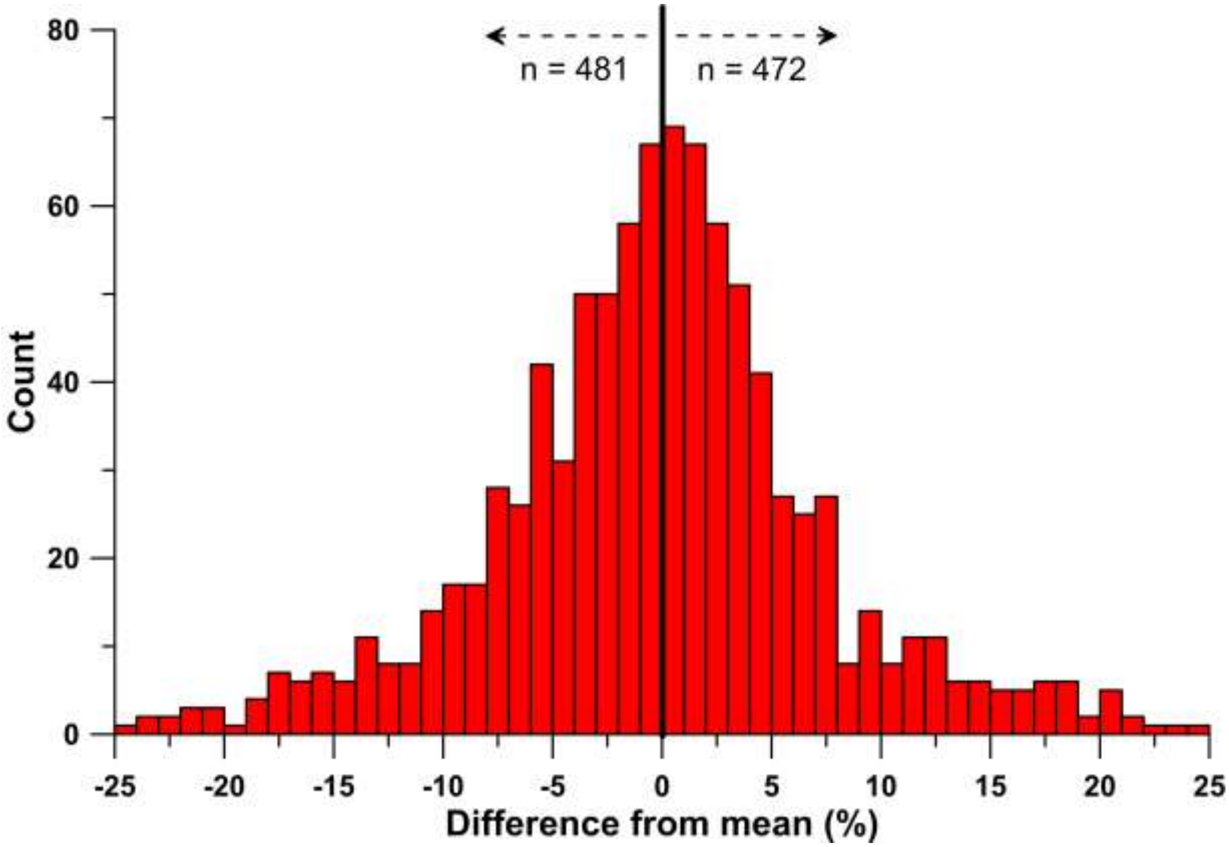

Supplementary Figure 7. Histogram showing the deviation of boulder ages from the mean age on each moraine. The number of samples on either side of the mean is indicated at the top of the figure. Only moraines with three or more boulder ages were included. Boulders considered outliers by the original authors were excluded.

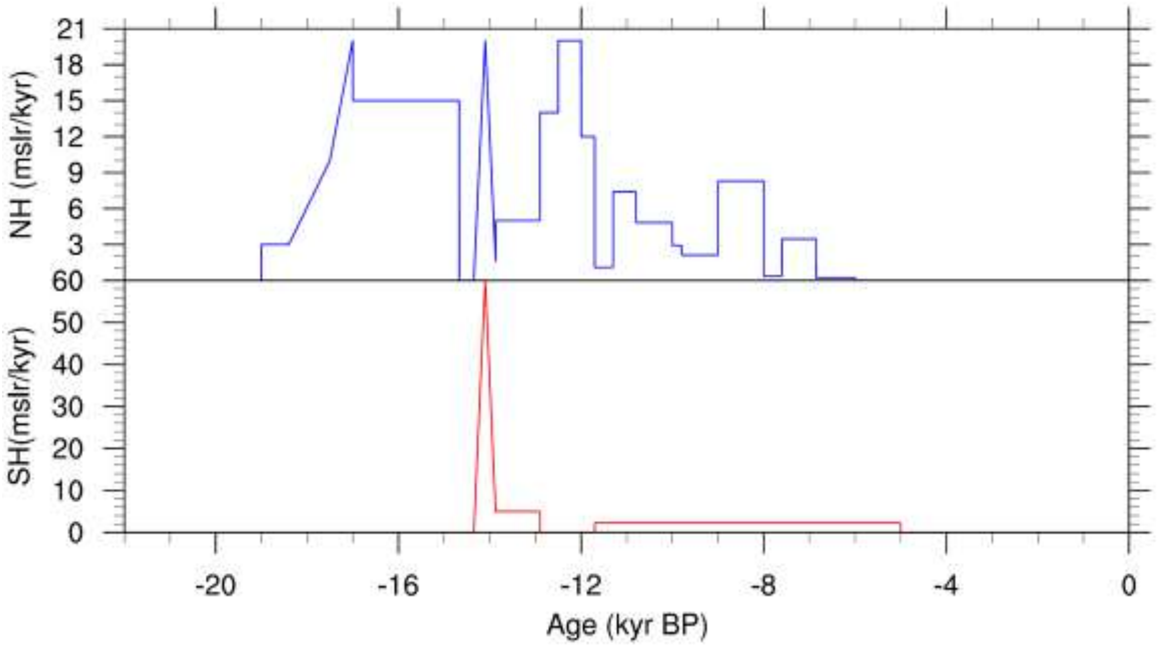

Supplementary Figure 8. Northern and Southern Hemisphere freshwater forcing used in the MOC and ALL simulations.

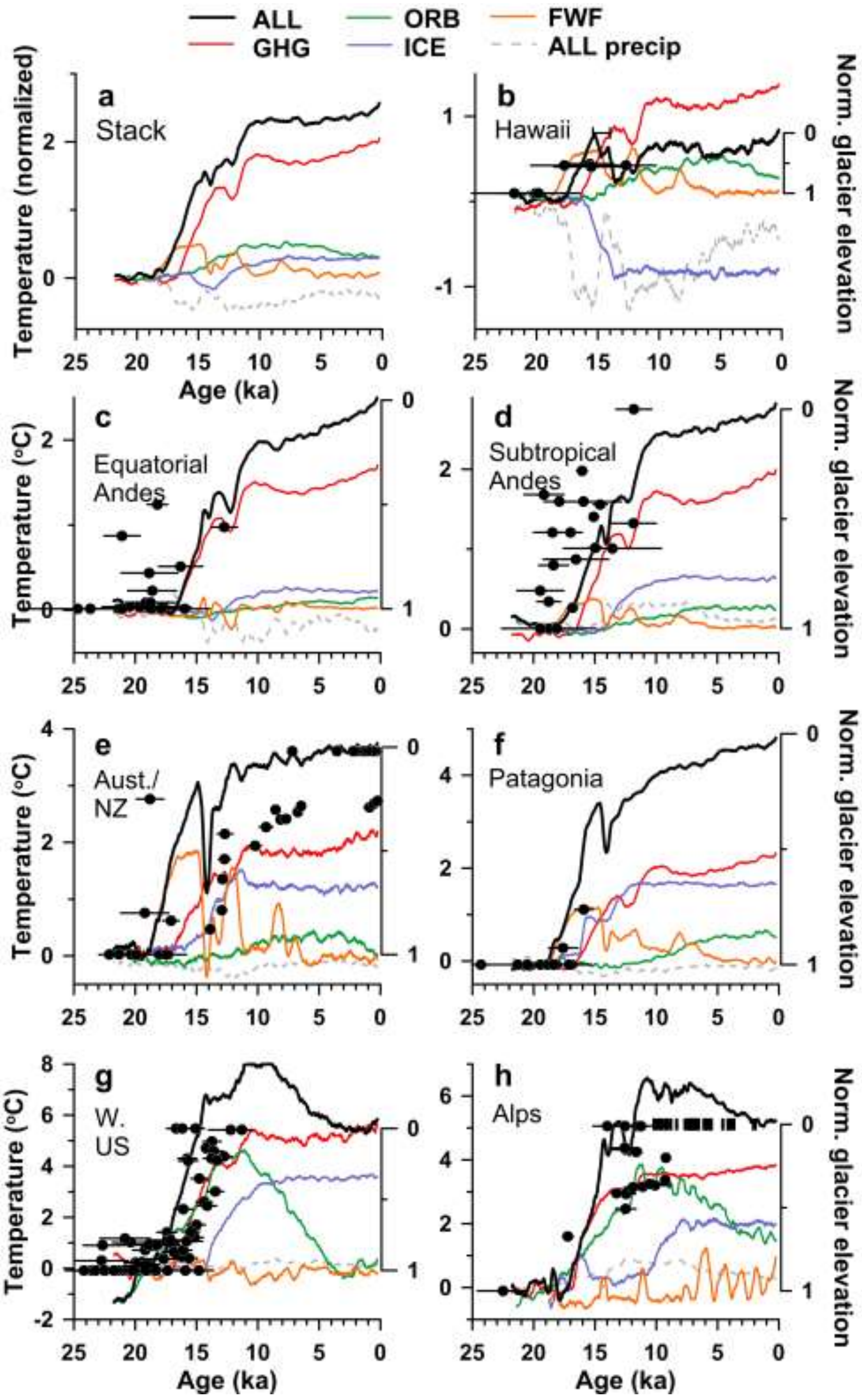

Supplementary Figure 9. As in Figure 3 in the manuscript, but here showing normalized glacier elevation changes rather than length changes.

Supplementary Table 1
Sea-level, high-latitude (SLHL) production rates for ^{10}Be using nuclide-specific LSD scaling

Study	SLHL Production Rate *(at g^{-1} y^{-1})*
Fenton et al.[4] – Norway	3.59 ± 0.18 *(1.7)*
Claude et al.[5] – Chironico landslide, Switzerland	3.94 ± 0.24 *(2.1)*
Ballantyne and Stone[6] – Scotland	4.01 ± 0.14 *(0.2)*
Kaplan et al.[7] – Puerto Bandera only, Patagonia	3.93 ± 0.10 *(1.3)*
Goehring et al.[8] – Norway	4.01 ± 0.12 *(3.4)*
Balco et al.[9] – NE North America	4.10 ± 0.20 *(1.8)*
Kelly et al.[10] – Peru *	4.13 ± 0.09 *(2.5)*
Putnam et al.[11] – New Zealand	3.77 ± 0.08 *(0.4)*
Young et al.[12] – Arctic	3.82 ± 0.13 *(0.3)*
Lifton et al.[13] – Lake Bonneville	4.20 ± 0.09 *(1.1)*
Mean – All	*3.9 ± 0.2*
Mean – No Fenton+	***4.0 ± 0.1***

Notes

Production rates only considered significant to one decimal place. 2nd decimal place only carried for rounding. Numbers in parentheses and italics following each site production rate are reduced χ^2 values for the fit at each site. Erosion rate and surface shielding corrections taken from original studies.

* Samples Q-47, Q-48 removed from dataset following Kelly et al.[10]. Samples Q-40a, Q-44a, and Q-83a also removed to prevent circularity since they were used to calculate surface erosion rates used for the other samples in the study. Including the latter 3 samples does not significantly change the site production rate (4.09 ± 0.09 ^{10}Be at g^{-1} y^{-1}). Assume erosion rates as stated in Kelly et al.[10]

\+ Production rate from Fenton et al.[4] (including all corrections from original study) fails Chauvenet's critierion[14], and thus is considered an outlier for this dataset.

Supplementary Table 2
SLHL production rates for ^{3}He using nuclide-specific LSD scaling

Study	SLHL Production Rate *(at g^{-1} y^{-1})*
Ackert et al.[15] – Patagonia*	127.6 ± 1.7 *(19.3)*
Amidon et al.[16] – Lake Bonneville *(pyroxene)*	125.1 ± 10.6 *(0.2)*
Blard et al.[17] – Mt. Etna*	131.4 ± 3.7 *(15.7)*
Blard et al.[17] – Hawaii*	149.9 ± 7.5 *(1.6)*
Blard et al.[18] – Altiplano	132.5 ± 4.1 *(2.0)*
Cerling and Craig[19], Cerling[20] – Western U.S. and France*	116.9 ± 3.5 *(4.3)*
Dunai and Wijbrans[21] – Canary Islands*	115.5 ± 13.1 *(0.1)*
Kurz et al.[22] – Hawaii*	134.6 ± 13.2 *(7.4)*
Goehring et al.[23] – Lake Bonneville*	118.8 ± 2.1 *(18.2)*
Licciardi et al.[24] – Western U.S.*	106.8 ± 4.3 *(1.1)*
Licciardi et al.[25] – Iceland*	114.4 ± 5.3 *(5.6)*
Foeken et al.[26] – Cape Verde Islands	94.7 ± 4.6 *(2.5)*
Mean	***122 ± 14***

Notes

Production rates only considered significant to whole numbers. First decimal place only carried for rounding. Numbers in parentheses and italics following each site production rate are reduced χ^2 values for the fit at each site. Erosion rate and surface shielding corrections taken from original studies

* Included in Goehring et al.[23]

Supplementary Note 1

Excluding Asian data

Cosmogenic ages from Asia were excluded from our analysis for two reasons. First, moraine boulder ages from this region tend to exhibit relatively large scatter and there is thus greater uncertainty in the timing of glacier fluctuations there. For instance, Heyman[41] synthesized 1855 ages on 113 moraines from the Tibetan Plateau and surrounding regions and found that more than half have uncertainties >15%, with many exceeding 30%. Second, most moraines dated in this region are from different valleys, and thus the retreat of individual glaciers from maximum to minimum extents cannot be scaled into the normalized units that we use to compare deglaciations across different regions and to potential forcings. For example, in Heyman's[41] synthesis, only one valley has a 'Class A' moraine (those with uncertainties <15%) representing the local LGM (taken to be Marine Isotope Stage 2, or 30-18 ka) with an additional Class A deglacial-age moraine upvalley.

Supplementary Note 2

Greenhouse gas forcing

While we focus on the relationship between glacier retreat and CO_2 forcing in the main text, a more complete treatment of greenhouse-gas forcing would also include the effects from CH_4 and N_2O. Nonetheless, our approximation is justified since CO_2 contributed nearly 80% of the combined radiative forcing from these gases, and CO_2 forcing rose to within 10% of its preindustrial value by 11 ka.

Supplementary References

1 Marcott, S. A. *et al.* Centennial-scale changes in the global carbon cycle during the last deglaciation. *Nature* **514**, 616-619, doi:10.1038/nature13799 (2014).

2 McManus, J. F., Francois, R., Gherardi, J. M., Keigwin, L. D. & Brown-Leger, S. Collapse and rapid resumption of Atlantic meridional circulation linked to deglacial climate changes. *Nature* **428**, 834-837 (2004).

3 Lambeck, K., Rouby, H., Purcell, A., Sun, Y. & Sambridge, M. Sea level and global ice volumes from the Last Glacial Maximum to the Holocene. *Proceedings of the National Academy of Sciences* **111**, 15296-15303, doi:10.1073/pnas.1411762111 (2014).

4 Fenton, C. R. *et al.* Regional 10Be production rate calibration for the past 12 ka deduced from the radiocarbon-dated Grøtlandsura and Russenes rock avalanches at 69° N, Norway. *Quaternary Geochronology* **6**, 437-452, doi:http://dx.doi.org/10.1016/j.quageo.2011.04.005 (2011).

5 Claude, A. *et al.* The Chironico landslide (Valle Leventina, southern Swiss Alps): age and evolution. *Swiss J Geosci* **107**, 273-291, doi:10.1007/s00015-014-0170-z (2014).

6 Ballantyne, C. K. & Stone, J. O. Did large ice caps persist on low ground in north-west Scotland during the Lateglacial Interstade? *Journal of Quaternary Science* **27**, 297-306, doi:10.1002/jqs.1544 (2012).

7 Kaplan, M. R. *et al.* In-situ cosmogenic 10Be production rate at Lago Argentino, Patagonia: Implications for late-glacial climate chronology. *Earth and Planetary Science Letters* **309**, 21-32, doi:http://dx.doi.org/10.1016/j.epsl.2011.06.018 (2011).

8 Goehring, B. M. *et al.* Late glacial and holocene 10Be production rates for western Norway. *Journal of Quaternary Science* **27**, 89-96, doi:10.1002/jqs.1517 (2012).

9 Balco, G. *et al.* Regional beryllium-10 production rate calibration for late-glacial northeastern North America. *Quaternary Geochronology* **4**, 93-107, doi:10.1016/j.quageo.2008.09.001 (2009).

10 Kelly, M. A. *et al.* A locally calibrated, late glacial 10Be production rate from a low-latitude, high-altitude site in the Peruvian Andes. *Quaternary Geochronology*, doi:http://dx.doi.org/10.1016/j.quageo.2013.10.007 (2013).

11 Putnam, A. E. *et al.* In situ cosmogenic 10Be production-rate calibration from the Southern Alps, New Zealand. *Quaternary Geochronology* **5**, 392-409, doi:10.1016/j.quageo.2009.12.001 (2010).

12 Young, N. E., Schaefer, J. M., Briner, J. P. & Goehring, B. M. A 10Be production-rate calibration for the Arctic. *Journal of Quaternary Science* **28**, 515-526, doi:10.1002/jqs.2642 (2013).

13 Lifton, N. *et al.* In situ cosmogenic nuclide production rate calibration for the CRONUS-Earth project from Lake Bonneville, Utah, shoreline features. *Quaternary Geochronology*, doi:http://dx.doi.org/10.1016/j.quageo.2014.11.002 (2015).

14 Bevington, P. R. & Robinson, D. K. *Data Reduction and Error Analysis for the Physical Sciences*. Second edn, (WCB/McGraw-Hill, 1992).

15 Ackert, J. R. P., Singer, B. S., Guillou, H., Kaplan, M. R. & Kurz, M. D. Long-term cosmogenic 3He production rates from 40Ar/39Ar and K-Ar dated Patagonian lava flows at 47[deg]S. *Earth and Planetary Science Letters* **210**, 119-136 (2003).

16 Amidon, W. H. & Farley, K. A. Cosmogenic 3He production rates in apatite, zircon and pyroxene inferred from Bonneville flood erosional surfaces. *Quaternary Geochronology* **6**, 10-21, doi:http://dx.doi.org/10.1016/j.quageo.2010.03.005 (2011).

17 Blard, P. H. *et al.* Cosmogenic 3He production rates revisited from evidences of grain size dependent release of matrix-sited helium. *Earth and Planetary Science Letters* **247**, 222-234 (2006).

18 Blard, P. H. *et al.* Cosmogenic 3He production rate in the high tropical Andes (3800 m, 20°S): Implications for the local last glacial maximum. *Earth and Planetary Science Letters* **377–378**, 260-275, doi:http://dx.doi.org/10.1016/j.epsl.2013.07.006 (2013).

19 Cerling, T. E. & Craig, H. Cosmogenic 3He production rates from 39oN to 46oN latitude, western USA and France. *Geochimica et Cosmochimica Acta* **58**, 249-255 (1994).

20 Cerling, T. E. Dating geomorphic surfaces using cosmogenic ^{3}He. *qr* **33**, 148-156 (1990).

21 Dunai, T. J. & Wijbrans, J. R. Long-term cosmogenic 3He production rates (152 ka-1.35Ma) from 40Ar/39Ar dated basalt flows at 29 degrees N lattitude. *Earth and Planetary Science Letters* **176**, 147-156 (2000).

22 Kurz, M. D., Colodner, D., Trull, T. W., Moore, R. B. & O'Brien, K. Cosmic ray exposure dating with in situ produced cosmogenic ^{3}He: Results from young Hawaiian lava flows. *Earth and Planetary Science Letters* **97**, 177-189 (1990).

23 Goehring, B. M. *et al.* A reevaluation of in situ cosmogenic 3He production rates. *Quaternary Geochronology* **5**, 410-418, doi:10.1016/j.quageo.2010.03.001 (2010).

24 Licciardi, J. M., Kurz, M. D., Clark, P. U. & Brook, E. J. Calibration of cosmogenic ^{3}He production rates from Holocene lava flows in Oregon, USA, and effects of the Earth's magnetic field. *Earth and Planetary Science Letters* **172**, 261-271 (1999).

25 Licciardi, J. M., Kurz, M. D. & Curtice, J. M. Cosmogenic 3He production rates from Holocene lava flows in Iceland. *Earth and Planetary Science Letters* **246**, 251-264 (2006).

26 Foeken, J. P. T., Stuart, F. M. & Mark, D. F. Long-term low latitude cosmogenic 3He production rate determined from a 126 ka basalt from Fogo, Cape Verdes. *Earth and Planetary Science Letters* **359–360**, 14-25, doi:http://dx.doi.org/10.1016/j.epsl.2012.10.005 (2012).
